# Integrating Venom Peptide Libraries Into a Phylogenetic and Broader Biological Framework

**DOI:** 10.3389/fmolb.2022.784419

**Published:** 2022-02-21

**Authors:** Kevin Chase, Maren Watkins, Helena Safavi-Hemami, Baldomero M. Olivera

**Affiliations:** ^1^ School of Biological Sciences, University of Utah, Salt Lake City, UT, United States; ^2^ Department of Biomedical Sciences, University of Copenhagen, Copenhagen, Denmark; ^3^ Department of Biochemistry, University of Utah, Salt Lake City, UT, United States

**Keywords:** turripeptides, conoidean venoms, phylogenetics, peptide libraries, *Turris*, *Purpuraturris*

## Abstract

The venomous marine snails are conventionally divided into three groups, the cone snails (family Conidae), the auger snails (family Terebridae) and the turrids (formerly all assigned to a single family, Turridae). In this study, a library of venom peptides from species conventionally assigned to the genus *Turris* was correlated to a phylogenetic analysis. Nucleotide sequences of multiple genes from transcriptomes were used to assess the phylogenetic relationships across a diverse set of species. The resulting tree shows that as conventionally defined, the conoidean genus *Turris*, is polyphyletic. We describe a new genus, *Purpuraturris gen. nov.*, that comprises the outlier species. In addition to morphological distinctions, molecular data reveal that this group is divergent from *Turris sensu stricto*. The correlation between phylogenetic information and a family of peptide sequences was used to highlight those peptides mostly likely to be unique and intimately associated with biological diversity. The plethora of peptide sequences available requires two prioritization decisions: which subset of peptides to initially characterize, and after these are characterized, which to comprehensively investigate for potential biomedical applications such as drug developments.

**Life Science Identifiers:** urn:lsid:zoobank.org; pub: 60D46561-28F0-4C39-BAC4-66DC8B4EAEA4

## Introduction

Evolution has crafted an exquisite library of bioactive peptides expressed in the venomous species of the world. This vast diversity of bioactive peptides has long been recognized as a tremendous pharmacological resource (Lewis and Garcia, 2003; Chen et al., 2018; Pennington et al., 2018). Historically, obtaining a single peptide sequence from individual specimens was so laborious that almost any peptide with a known primary sequence was heavily investigated. Recent technological advances have made identifying primary peptide sequences facile, placing nearly the entire library at our fingertips. As a result, the available sequences of venom peptides are undergoing an explosive growth phase. This creates a general problem for the field of venom research—the ever-expanding catalog of peptide sequences has far outstripped our present capacity to elucidate the mechanisms that underlie their activity. For this reason, prioritizing which venom peptide to investigate is becoming increasingly important. Here we demonstrate that leveraging the phylogeny of common housekeeping genes against the phylogeny of a family of peptide sequences can highlight which peptide sequences are most likely to be unique and intimately associated with biological diversity. By organizing venom peptide sequences using this phylogenetic framework, the ever-expanding venom peptide libraries can be correlated and assembled into groups that reflect the underlying biology of the animals.

The focus of this paper is a library of venom peptides generated by species traditionally assigned to the genus *Turris*, the type genus of the family Turridae (turrids). Turrids are one of the lesser-known venomous marine snails in the superfamily Conoidea. Compared to some other groups in the superfamily, such as the cone snails (family Conidae), the turrids are a relatively neglected lineage. We illustrate how the correlation between phylogenetic information and peptide sequences obtained from multiple species in a turrid lineage provides useful insights into what otherwise might seem like a confusing group of peptides.

**FIGURE 1:**
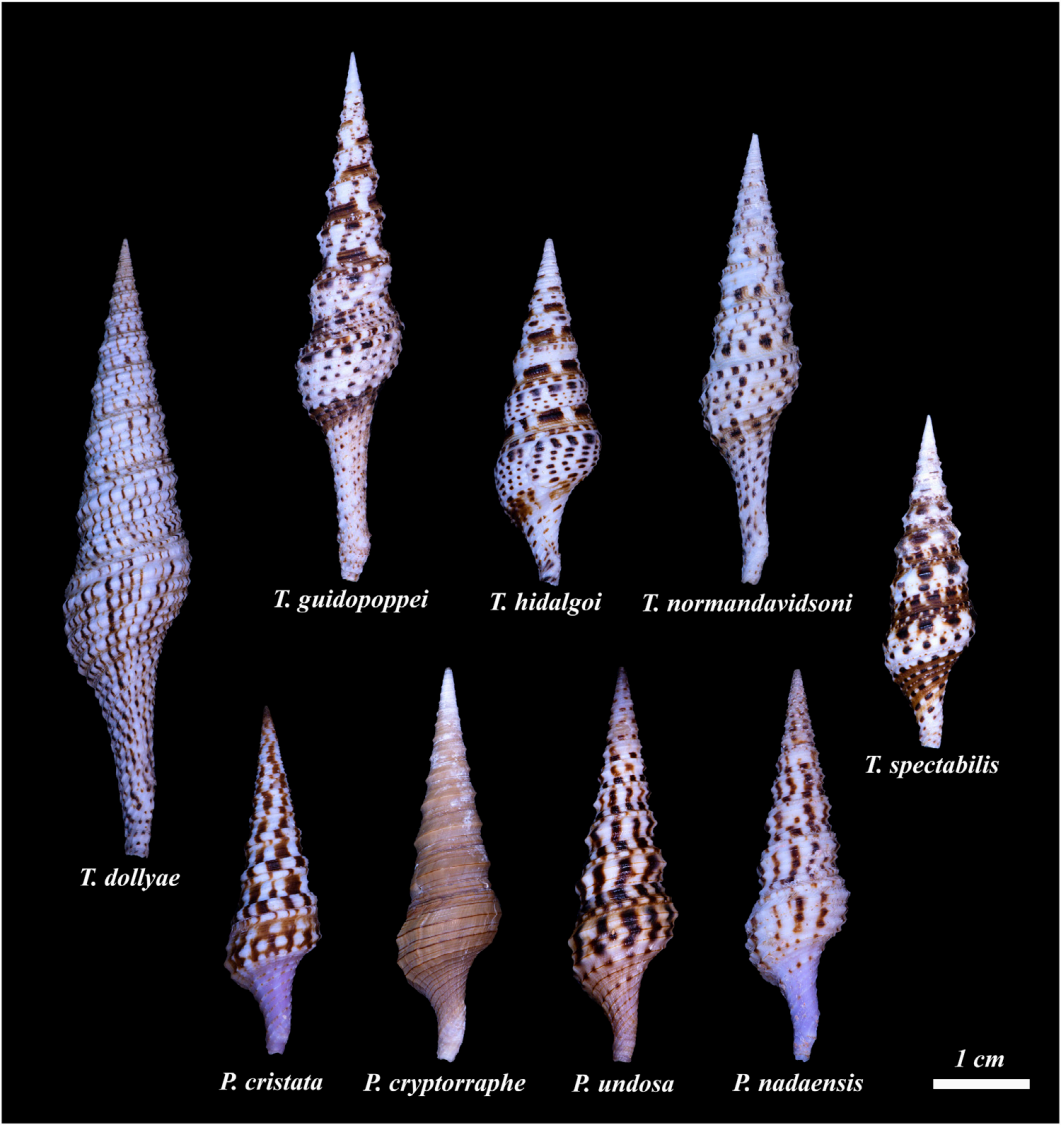
The nine species conventionally assigned to the genus *Turris* for which a venom gland transcriptome is available are shown. Top row left to right: *Turris dollyae*, *Turris guidopoppei*, *Turris hidalgoi*, *Turris normandavidsoni*, *Turris spectabilis*. The species shown on the lower row were assigned to the genus *Turris*, but as will be demonstrated through the data obtained in this work, they belong to a new genus, *Purpuraturris*. From left to right: *Purpuraturris cristata*; *Purpuraturris cryptorraphe*; *Purpuraturris undosa*; *Purpuraturris nadaensis*. The shells were photographed individually and the photographs were cropped to maintain the size of the shells relative to each other. The scale bar represents an approximate length of 1 cm (Photographed by Samuel Espino.)

**FIGURE 2:**
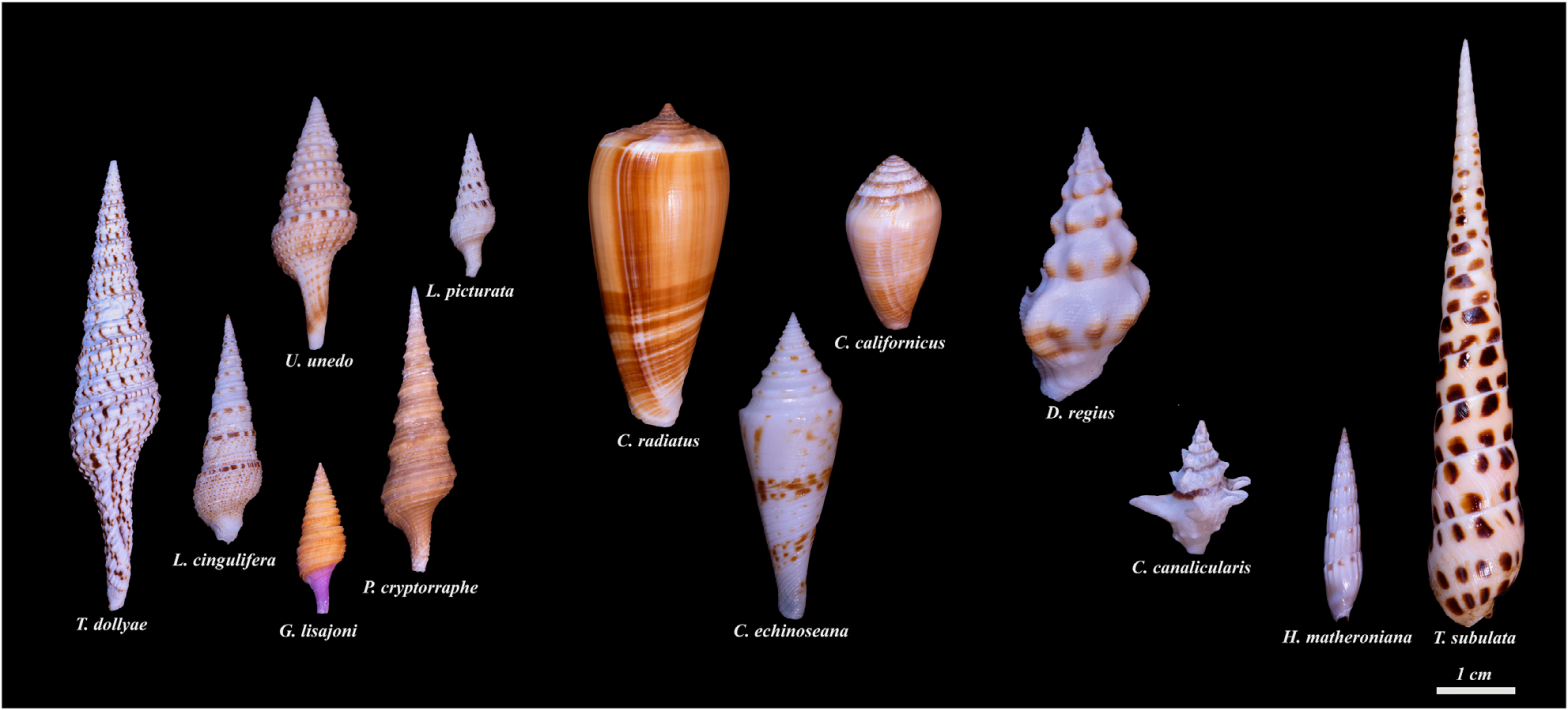
Conoidean families and genera included in the transcriptome-based phylogenetic tree. The six specimens on the left belong to the family Turridae, followed by three specimens in the family Conidae. The two rightmost specimens belong to the family Terebridae and the two next to them are in the family Drilliidae. Starting with the leftmost specimen, and going clockwise: Family Turridae: *Turris* (*Turris dollyae*); *Iotyrris* (*Iotyrris cingulifera*); *Unedogemmula* (*Unedogemmula unedo*); *Lophiotoma* (*Lophiotoma picturata*); *Purpuraturris* (*Purpuraturris cryptorraphe*); *Gemmula* (*Gemmula lisajoni*). Family Conidae: *Conus* (*Conus radiatus*); *Californiconus* (*Californiconus californicus*); *Conasprella* (*Conasprella ichnoseana*). Family Drilliidae: *Drillia* (*Drillia regia*); *Clavus* (*Clavus canalicularis*). Family Terebridae: *Hastula* (*Hastula matheroniana*); *Terebra* (*Terebra subulata*). The shells were photographed individually and the photographs were cropped to maintain the size of the shells relative to each other. The scale bar represents an approximate length of 1 cm (Photographed by Samuel Espino).

The Superfamily Conoidea (Figures 1, 2) is a biodiverse lineage of predatory marine snails, the vast majority of which are venomous (Olivera et al., 2014). The best known are the cone snails (family Conidae). A second familiar group are the auger snails (family Terebridae), which are highly adapted to sandy marine habitats (Holford et al., 2009). Traditionally, all other conoideans were included in the family Turridae (Bouchet, 1990), but it became apparent that this was a heterogenous group. In recent years, the family Turridae has been restricted only to the conoidean lineages more closely-related to the type species, *Turris babylonia* (the Tower of Babel turrid); most other species formerly included in Turridae are now assigned to other families (Puillandre et al., 2008; Bouchet et al., 2011), many of which were formerly regarded as subfamilies of Turridae. Even in its reduced form, the taxonomy and phylogeny of the family has presented several confusing taxonomic and phylogenetic problems [perhaps aptly symbolized by having the Tower of Babel turrid as type species (Olivera et al., 2010)]. The subfamilies and genera within the family Turridae have not been treated in a consistent manner, and very often, even well-known species have been assigned to different genera at various times (Horton et al., 2021). These types of taxonomic problems are generally true of the more biodiverse lineages of venomous animals.

In this study, we have used a new approach to investigate the phylogeny of the Turridae, with a specific focus on the genus *Turris*. Because recent advances in transcriptome sequencing have made it possible to sequence the venom gland of most if not all available venomous species, we have assessed phylogenetic relationships within the family Turridae and other conoidean families based on the venom gland transcriptomes of 35 conoidean species. The results presented here confirm the finding by Fedosov et al. (2011) that the type genus of the family Turridae, the genus *Turris*, is not monophyletic. The species in the genus can be divided into two major groups, those species most closely related to the type, *Turris babylonia*, and a second group, which on the basis of our phylogenetic analysis should not be assigned to *Turris*. The solution we propose is the creation of a new genus, *Purpuraturris gen. nov.*, with *Purpuraturris cryptorrhaphe* (formerly *Turris cryptorrhaphe*) as the type species.

**FIGURE 3:**
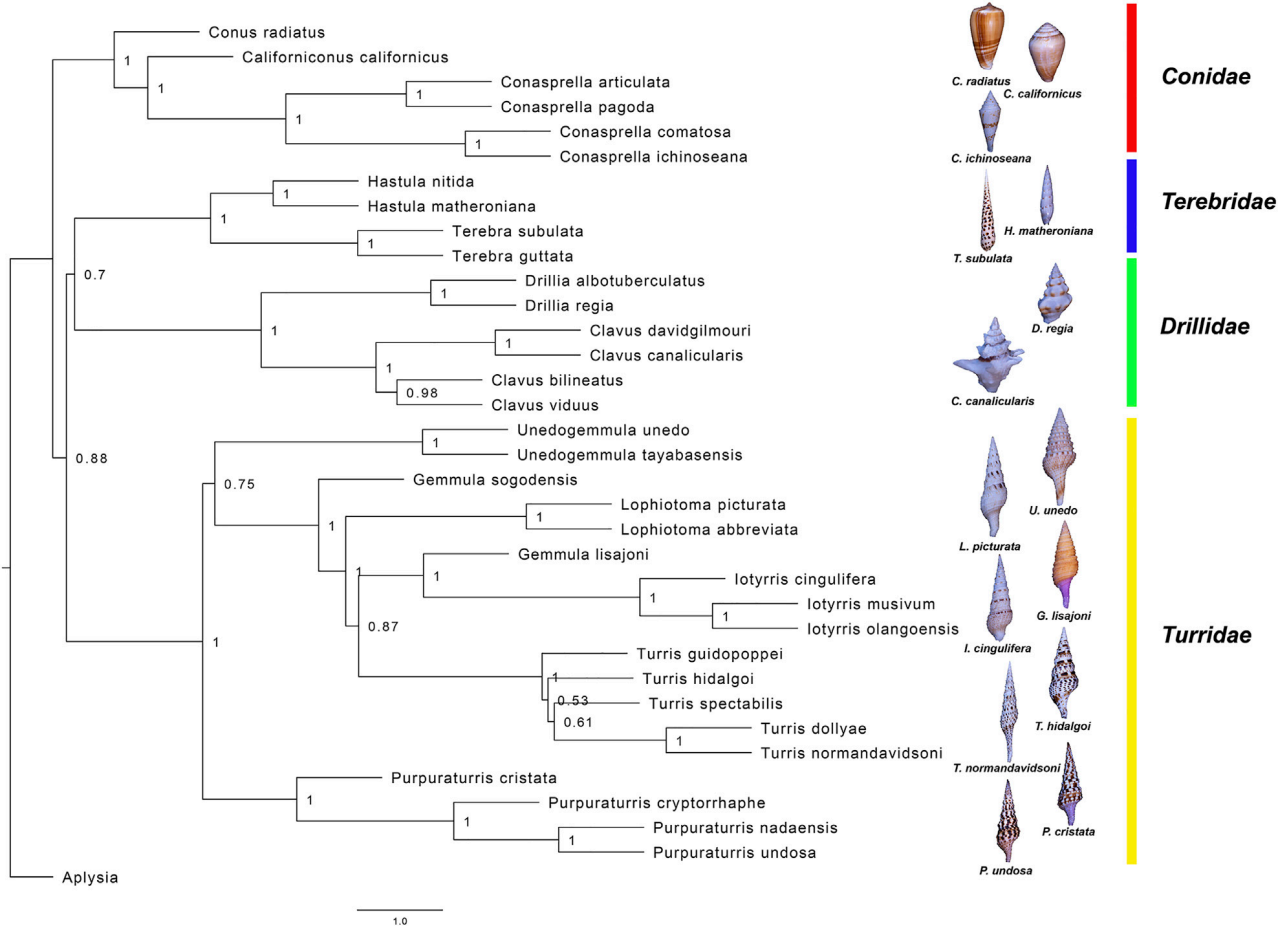
Maximum likelihood phylogenetic tree based on transcriptome sequences from 66 contigs with blast identity to common housekeeping genes. The tree shown here represents the consensus tree from the 66 individual gene trees. The values at the nodes indicate the fraction of individual gene trees that support that node. The lengths of the terminal branches are undetermined. Conoidean families are labelled and representative images shown.

Phylogenetic analysis based on the nucleotide sequences of a set of common genes that are expressed in the transcriptomes of these conoidean molluscs yields a surprisingly robust phylogeny (Figure 3). This is consistent with recent findings on the use of gene fragments obtained by exon capture for the phylogenetic reconstruction of the conoidean tree (Abdelkrim et al., 2018). Our results suggest that the family Turridae is the sister group of the clade which includes both family Terebridae and family Drillidae, and that the group of species formerly in *Turris* that do not belong in that genus fall in a separate major branch of the family Turridae. The phylogenetic framework we established for *Turris* was then used to analyze a family of venom peptides, two closely-related divisions in the P-like Turripeptide Superfamily. The analysis of this family of peptides was correlated to the phylogenetic information; the results are presented and discussed.

## Results

### Phylogenetic Analysis of Superfamily Conoidea Based on Transcriptomes

Our laboratories have carried out an extensive analysis of the transcriptomes of venom glands of cone snails (family Conidae) (Safavi-Hemami et al., 2016; Li et al., 2017; Robinson et al., 2017; Li et al., 2018; Lu et al., 2020). This information has greatly facilitated the characterization of bioactive venom components. We have also analyzed a number of taxa in the superfamily Conoidea which are not cone snails; many of these belong to the family Turridae (Figures 1–3, Supplementary Tables S1, S2). However, species in some other conoidean families, including Terebridae and Drilliidae, have also been analyzed (see Figures 2, 3).

Using these datasets, we have developed a method for phylogenetic analysis based on common transcripts recovered from transcriptomic data; a detailed description of the procedures and protocols is provided in the Methods section. This approach should be applicable to any molluscan group. The results of this analysis, shown in Figure 3, include nine different species conventionally assigned to the genus *Turris*. The results clearly demonstrate that *Turris*, as conventionally defined, is polyphyletic, and that a subset of species is only distantly related to the major group. We describe a new genus, *Purpuraturris gen. nov.*, to include this subset of outlier species (see Appendix 1). The phylogenetic tree in Figure 3 demonstrates that the family Turridae has two major divisions. The four species (*cryptorraphe*, *cristata*, *undosa*, *nadaensis*) formerly assigned to *Turris* are not in the same branch as the other five species in *Turris* (*guidopoppei*, *hidalgoi*, *spectabilis*, *dollyae, normandavidsoni*).

**FIGURE 4:**
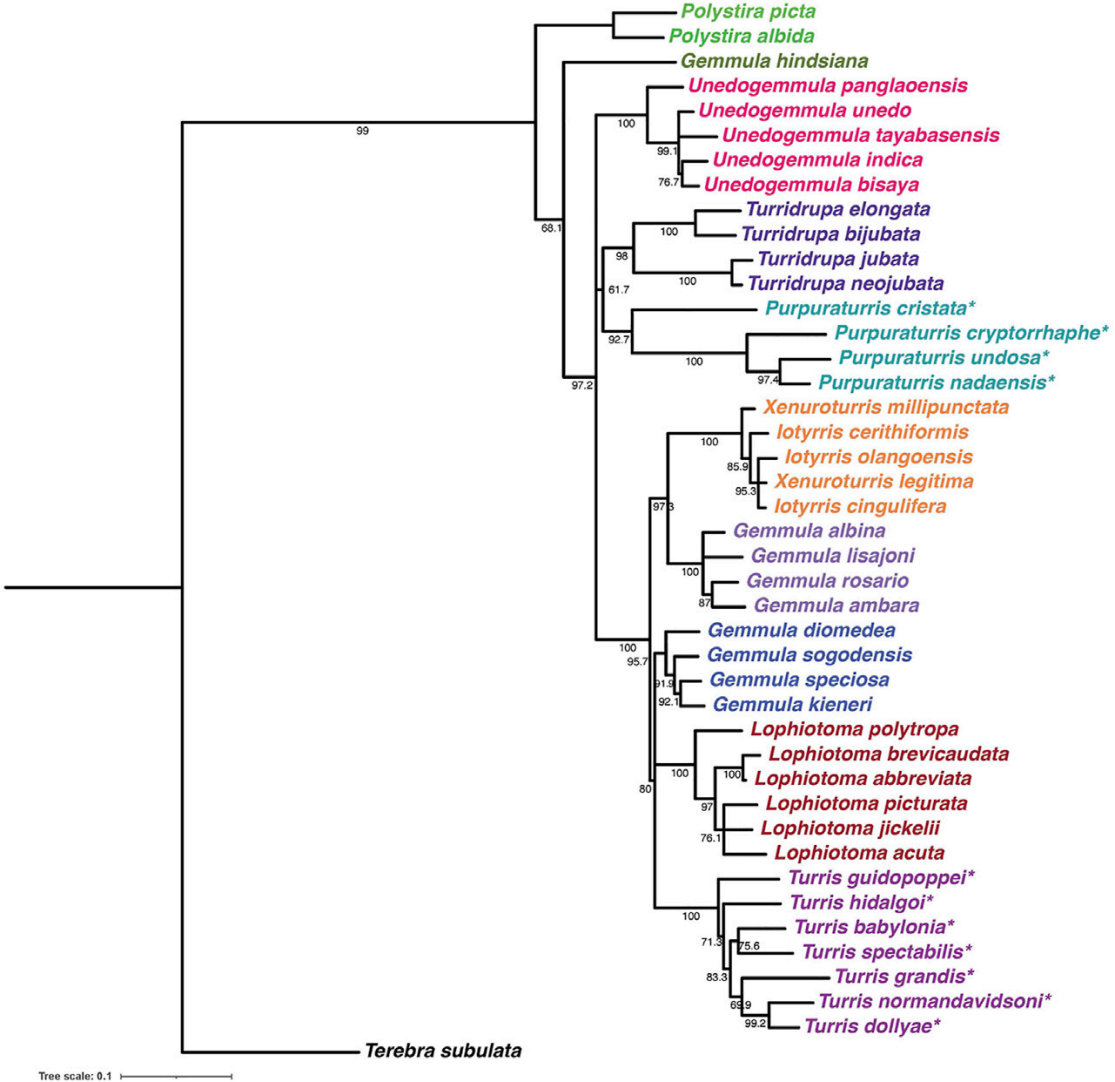
Maximum likelihood phylogenetic tree of Turridae with bootstrap support values. The tree uses standard phylogenetic markers (the GenBank accession numbers and publication references are listed in Supplementary Table S5) and was constructed as described under Methods. We have marked with an asterisk the species that were formerly assigned to the genus *Turris*; as shown in the tree, these are widely separated into two groups, *Turris*, colored purple (now all assigned to the genus *Turris senso stricto*) and the turquoise branch (assigned to the new genus *Purpuraturris gen. nov.*) The tree illustrates some of the remaining taxonomic problems in Turridae: note that species conventionally assigned to *Gemmula* fall in multiple branches, while there is no separation between *Xenuroturris* and *iotyrris*.

The proposed new genus includes these four species conventionally assigned to *Turris*, but for which the genetic data are incompatible with that assignment. The molecular results suggest that these species are unrelated to their former congeners in the genus *Turris*. Additionally, as a group these species are morphologically distinct and share a number of characters that justify creation of a new genus. Because they have some morphological characters similar to *Turris* (i.e., the position of the slit, the thin linear cleft in the margin of the aperture), but also have differentiating characters, notably a distinctive purplish color, particularly within the aperture, we have chosen the name *Purpuraturris gen. nov*. The type species of the new genus is *Purpuraturris cryptorrhaphe* (formerly *Turris cryptorrhaphe*). The transcriptomic data indicate that *Purpuraturris nadaensis, Purpuraturris undosa* and *Purpuraturris cristata* are congeners. This taxonomic placement is supported by the phylogenetic tree constructed using standard genetic markers (Figure 4). A conventional taxonomic description of the new genus is provided in Appendix 1.

### P-Like Turripeptides

**FIGURE 5:**
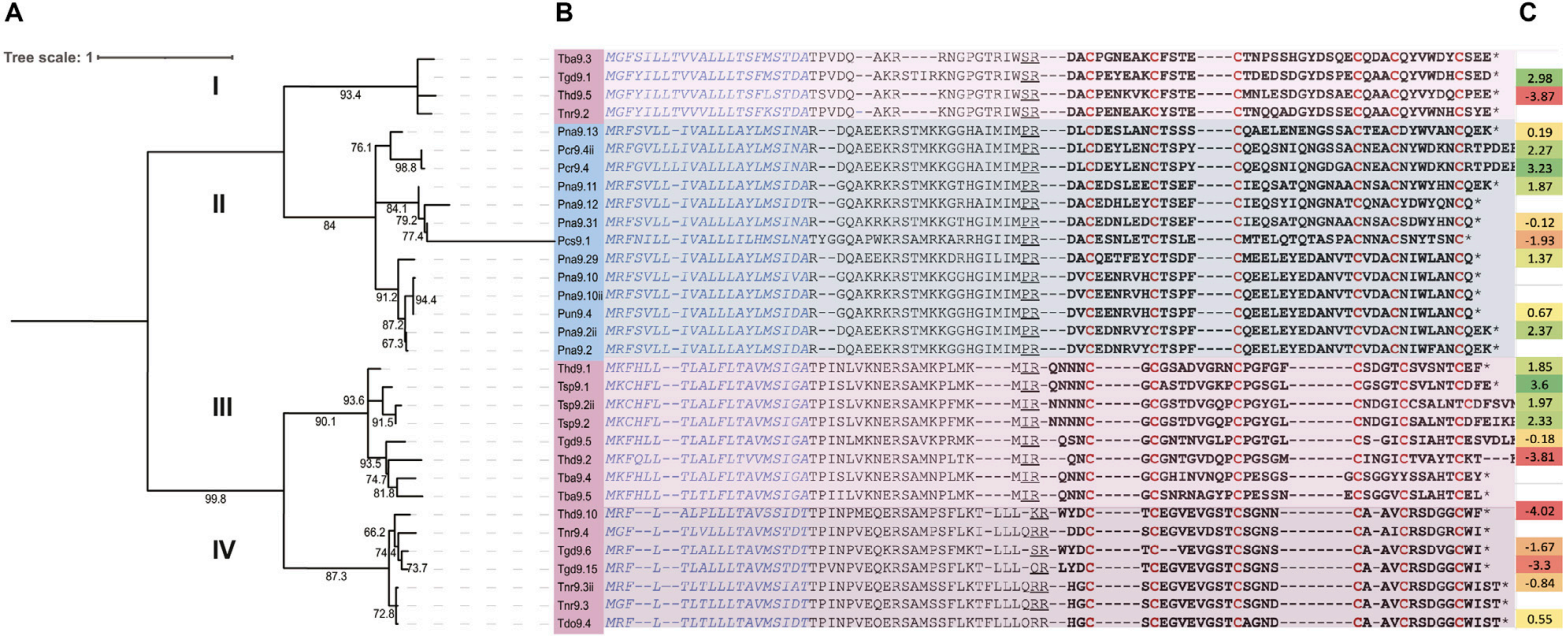
Maximum likelihood phylogenetic tree with bootstrap support values showing the major divisions of P-like turripeptides identified in *Turris* and *Purpuraturris* species into four discrete branches, labeled Clades I-IV. *Purpuraturris* is found only in Clade II. **(A)** phylogenetic tree, **(B)** multiple sequence alignment used to generate the tree, **(C)** Expression levels of turripeptides identified in transcriptomes relative to the mean expression of HKG in the same transcriptome [log_2_ (turripeptide_TPM_/HKG_meanTPM_) of the housekeeping fold change].

The specific family of venom peptides that is the focus of this study are venom peptides from species formerly assigned to the genus *Turris*; these belong to a group known as the P-like turripeptides (Watkins et al., 2006). This nomenclature is derived from the number and arrangement of cysteine residues in these peptides, which are shared with the P-superfamily of conotoxins (Robinson and Norton, 2014). A description of the nomenclature adopted for turrid venom peptides is provided in Appendix 2. Based on their signal sequences, propeptides, and cysteine loop sizes, these can either be regarded as a single superfamily of venom peptides, or as two closely-related superfamilies (see PII and PIII superfamilies, Supplementary Table S1). This superfamily was chosen due to the presence of multiple venom peptides from both *Turris* and *Purpuraturris*. The predicted mature peptide sequences are shown in Table 1, while a tree that shows how the sequences are related, along with the alignment of the precursor sequences used to generate the tree are presented in Figure 5. The precursor sequences are listed in Supplementary Table S1, divided into P-like turripeptide superfamily PII, consisting of Clades I and II in Figure 5, and P-like turripeptide superfamily PIII, consisting of Clades III and IV in Figure 5.

**TABLE 1:** Predicted mature P-like turripeptide sequences.

Species	Toxin name	Predicted mature peptide sequence
Clade I P-like turripeptides		
*** *** *Turris babylonia*	Tba9.3	DACPGNEAKCFSTECTNPSSHGYDSQECQDACQYVWDYCSEE
*** *** *Turris guidopoppei*	Tgd9.1	DACPEYEAKCFSTECTDEDSDGYDSPECQAACQYVWDHCSED
*** *** *Turris hidalgoi*	Thd9.5	DACPENKVKCFSTECMNLESDGYDSAECQAACQYVYDQCPEE
*** *** *Turris normandavidsoni*	Tnr9.2	DACPENEAKCYSTECTNQQADGYDSSECQAACQYVWNHCSYE
Clade II P-like turripeptides		
*** *** *Purpuraturris nadaensis*	Pnd9.13	DLCDESLANCTSSSCQAELENENGSSACTEACDYWVANCQE
*** *** *Purpuraturris cryptorrhaphe*	Pcr9.4ii	DLCDEYLENCTSPYCQEQSNIQNGSSACNEACNYWDKNCRTPDEEQ
*** *** *Purpuraturris cryptorrhaphe*	Pcr9.4	DLCDEYLENCTSPYCQEQSNIQNGDGACNEACNYWDKNCRTPDEEQ
*** *** *Purpuraturris nadaensis*	Pna9.11	ACEDSLEECTSEFCIEQSATQNGNAACNSACNYWYHNCQE
*Purpuraturris nadaensis*	Pna9.12	DACEDHLEYCTSEFCIEQSYIQNGNATCQNACYDWYQNCQ
*** *** *Purpuraturris nadaensis*	Pna9.31	DACEDNLEDCTSEFCIEQSATQNGNAACNSACSDWYHNCQ
*** *** *Purpuraturris cristata*	Pcs9.1	DACESNLETCTSLECMTELQTQTASPACNNACSNYTSNC
*** *** *Purpuraturris nadaensis*	Pna9.29	DACQETFEYCTSDFCMEELEYEDANVTCVDACNIWLANCQ
*Purpuraturris nadaensis*	Pna9.10	DVCEENRVHCTSPFCQEELEYEDANVTCVDACNIWLANCQ
*** *** *Purpuraturris nadaensis*	Pna9.10ii	﻿DVCEENRVHCTSPFCQEELEYEDANVTCVDACNIWLANCQ
*** *** *Purpuraturris undosa*	Pun9.4	DVCEENRVHCTSPFCQEELEYEDANVTCVDACNIWLANCQ
*** *** *Purpuraturris nadaensis*	Pna9.2ii	DVCEDNRVYCTSPFCQEELEYEDANVTCVDACNIWLANCQE
*** *** *Purpuraturris nadaensis*	Pna9.2	DVCEDNRVYCTSPFCQEELEYEDANVTCVDACNIWFANCQE
Clade III P-Clade like turripeptides		
*** *** *Turris hidalgoi*	Thd9.1	QNNNCGCGSADVGRNCPGFGFCSDGTCSVSNTCEF
*** *** *Turris spectabilis*	Tsp9.1	QNNNCGCASTDVGKPCPGSGLCGSGTCSVLNTCDFE
*** *** *Turris spectabilis*	Tsp9.2ii	NNNNCGCGSTDVGQPCPGYGLCNDGICSALNTCDFSVN
*** *** *Turris spectabilis*	Tsp9.2	NNNNCGCGSTDVGQPCPGYGLCNDGICSALNTCDFEI
*** *** *Turris guidopoppei*	Tgd9.5	QSNCGCGNTNVGLPCPGTGLCSGICSIAHTCESVDL
*** *** *Turris hidalgoi*	Thd9.2	QNCGCGNTGVDQPCPGSGMCINGICTVAYTCKT
*** *** *Turris babylonia*	Tba9.4	QNNCGCGHINVNQPCPESGSGCSGGYYSSAHTCEY
*** *** *Turris babylonia*	Tba9.5	QNNCGCSNRNAGYPCPESSNECSGGVCSLAHTCEL
Clade IV P-Like turripeptides		
*** *** *Turris hidalgoi*	Thd9.10	WYDCTCEGVEVGSTCSGNNCAAVCRSDGGCWF
*** *** *Turris normandavidsoni*	Tnr9.4	DDCSCEGVEVDSTCSGNSCAAICRSDGRCWI
*** *** *Turris guidopoppei*	Tgd9.6	WYDCTCVEVGSTCSGNSCAAVCRSDVGCWI
*** *** *Turris guidopoppei*	Tgd9.15	LYDCTCEGVEVGSTCSGNSCAAVCRSDGGCWI
*** *** *Turris normandavidsoni*	Tnr9.3ii	HGCSCEGVEVGSTCSGNDCAAVCRSDGGCWIST
*** *** *Turris normandavidsoni*	Tnr9.3	HGCSCEGVEVGSTCSGNDCAAVCRSDGGCWIST
*** *** *Turris dollyae*	Tdo9.4	HGCSCEGVEVGSTCAGNDCAAVCRSDGGCWIST

It is clear from this tree that the peptide sequences fall into discrete branches, which we have labeled Clades I–IV. It is notable that each branch of the tree either has species from *Turris* s.s. (as redefined above), or only species that have been removed from the genus *Turris* and reassigned to the proposed new genus, *Purpuraturris gen. nov*. None of the branches have a mixture of species from both genera. Thus, the grouping of several species in a given branch is consistent with and supportive of the phylogenetic trees shown in Figures 3, 4. The peptide tree reflects the phylogenetic relationships between the various taxa analyzed: the species in *Turris* are not closely-related to species in *Purpuraturris*, leading to the pattern of divergence in their venom peptides observed in Figure 5. The tree of peptide sequences has two major branches; in one major branch, there are two clades, with each subgroup containing sequences only from *Turris* s.s., or *Purpuraturris* species; in contrast, the second major branch has two clades, both having only species assigned to *Turris* whereas peptides from *Purpuraturris* are absent. Thus, a juxtaposition of the trees based on evolutionary relationships between species in Figures 3, 4, and on peptide sequence similarities in Figure 5 suggests that the divergence between the two major divisions of the P-like turripeptides shown in Figure 5 may have predated the divergence of *Purpuraturris* from *Turris*. Alternatively, it is possible that the divergence of Clades III and IV may have predated the divergence of Clade II (*Purpuraturris*) from Clade I (*Turris*) with a subsequent loss of these two lineages from *Purpuraturris*.

We note that the sequences of the mature peptides in the two major branches have strikingly divergent features. The two clades in the major branch found only in *Turris* species (Clades III and IV) have P-like turripeptides each with a single amino acid between the first two cysteine residues; in Clades I and II there are six amino acids between the first two cysteine residues, with the consensus sequence of: (E/P) EN (E/L) (A/E) X.

### Venom Peptide Libraries: From Families of Sequences to Experimental Strategies

The venom peptide family that is analyzed in Figure 5 and Table 1 has over 30 related sequences. In Figure 5, we have subdivided the major branches into four defined clades; the phylogenetic framework indicates that the peptides in Clade II should be considered separately from the peptides in Clade I, III and IV, since the divergence established by the phylogenetic trees in Figures 3, 4 indicate that these peptides may have evolved as part of an entirely different biological strategy, from two lineages of venomous animals that are long diverged. Nevertheless, Clades I, III and IV, still raise the issue of how to efficiently analyze this library of diverse peptides. Using phylogenetic methodology to compare sequences resulted in the discovery that peptides from *Turris* venoms that belong to the P-like turripeptide group fall into three clades; if sufficient resources were available, at least one peptide from each clade should be characterized, since it is likely that these may act through divergent mechanisms. Even within each clade of peptides, there are too many to comprehensively characterize. In the most complex group, Clade III, the mature peptide sequences can be clustered into two groups; the peptide sequences from *Turris hidalgoi* have representatives in each of these Clade III subgroups, i.e., Thd9.2 defining one group, and Thdg9.1 defining the second.

The use of a transcriptomic approach to building the phylogenetic tree automatically reveals relative expression levels (Figure 5C). If the expression levels of the two *T. hidalgoi* peptides in Clade III are compared, by far the most highly-expressed is Thd9.1. The high-expression level of Thd9.1 would more easily facilitate characterization and thus favor prioritization of this peptide for further study.

## Discussion

### Using Transcriptomics for the Analysis of Conoidean Venoms

In the superfamily Conoidea, the increasing number of comprehensive transcriptomic analyses of venom glands has made it possible to routinely generate native venom peptide libraries. This is particularly facile for conoidean venoms because of how venom peptide precursors are organized. The typical conoidean venom gland has a repertoire of approximately 200 different peptides encoded and expressed by epithelial cells lining the gland in each species. The accelerated evolution of these venom peptide genes results in the mature peptide regions being hypervariable, except for the cysteine residues which provide a conserved framework for genetically related peptides. Because the signal sequences are also very highly conserved, it is straightforward to identify related peptides in each conoidean lineage. In this study, we demonstrate how the transcriptomic analysis of one lineage can be used to identify a peptide family, and in addition, to reveal phylogenetic relationships. Until recently, this type of analysis was significantly more difficult, since obtaining a comprehensive transcriptome was much more cumbersome and expensive.

### Analysis of P-like Turripeptides

The peptide family we analyzed in this study was obtained from the venom glands of species conventionally assigned to a single genus, *Turris*. One previously characterized group of venom peptides in turrids are the P-like turripeptides (Heralde et al., 2008), which have six cysteine residues in a pattern characteristic of *Conus* P-superfamily peptides (Robinson and Norton, 2014) but which are otherwise unrelated. There is more than one gene superfamily that encodes peptides of this type in these species (Watkins et al., 2006) — in this study, we focused on a subset of P-like turripeptides with related signal sequences. The precursor sequences, which are the data used to predict sequences of the mature bioactive venom peptides, are shown in Figure 5 and Supplementary Table S1. In some cases, P-like turripeptide precursor sequences were obtained using PCR or sequencing of cDNA libraries rather than from transcriptomes, indicated in Supplementary Table S1 by asterisks. In a few cases, P-like turritoxin precursor sequences were obtained from a transcriptome of a specimen other than that used for the neutral marker analysis in Figure 3. Those sequences are included in Supplementary Table S1, indicated by double asterisks. Expression levels of all P-like turripeptides identified in transcriptomes relative to the mean expression of HKG in the same transcriptome are shown in Figure 5C and provide potential insight into the functional role of each peptide within a species. The species assigned to the genus *Turris sensu lato* for which a transcriptome was obtained and analyzed in this work are shown in Figure 1.

In addition to venom peptide genes, transcriptomic analysis also provides sequences for all of the other genes expressed in the venom glands, such as housekeeping genes and genes involved in the normal cellular processes required to produce the venom components (i.e., proteins involved in secretion and membrane trafficking). This sequence information was used to carry out a “neutral” phylogenetic analysis of the species that are simultaneously being analyzed for the identity of venom components. Thus, transcriptomic analysis leads to the easy identification of peptide families from a particular lineage of venomous animals, provides neutral estimates of the phylogenetic relationships between the species being analyzed, and establishes a robust basis for the relationship of that species group to other taxa for which similar transcriptomic data are available. The results of the phylogenetic analyses of the species shown in Figures 1, 2 are discussed in the following section; the relationship of the species in Figure 1 to other conoideans was assessed, using transcriptomic information from the genera illustrated in Figure 2.

### Phylogeny of Turrids

The phylogenetic tree in Figure 3, which was constructed using the transcriptomic approach described under Methods, includes four different conoidean families; all family assignments are firmly supported. The family Terebridae includes two genera, *Terebra*, represented by *Terebra subulata* and *Terebra guttata,* and *Hastula*, including *Hastula nitida* and *Hastula matheroniana*, that define two subbranches on the tree. Similarly, the family Drilliidae is represented by *Drillia* and *Clavus*, and the three genera in Conidae; *Californiconus* (*Conus californicus*), *Conasprella* and *Conus,* are well-supported in this phylogenetic analysis. Thus, the approach used is consistent with present generic assignments in the families Clavidae, Terebridae and Conidae (Bouchet et al., 2011).

In the family Turridae, species conventionally assigned to the genus *Turris* (shown in Figure 1) are split between two major branches of the family, with most species analyzed remaining in *Turris*, but some conventionally assigned to *Turris* have proven to be only distantly related. We have proposed a new genus, *Purpuraturris gen. nov.* (see the Appendix 1 and Figures 3, 4). The problem of proper generic assignments does not apply only to *Turris*, but also to other major genera in Turridae, such as *Gemmula* which could be addressed using similar methodologies in the future.

### A Biological Framework for Venom Peptide Libraries

Linking primary amino acid sequences of venom peptides to the phylogeny of the animals in which they evolved is a prerequisite for integrating the biochemical and molecular information obtained from the standard approach used in venom peptide research with the broader biology (and even ecology) of the venomous animals that evolved that library of peptides. The phylogenetic correlation provided for the P-like turripeptides in Figure 5 illustrates a strategy for the further definition and characterization of these peptides. We have shown that there are four major groups of peptides based on sequence similarities, one found in those species that we have moved to the new genus, *Purpuraturris gen. nov.*, and three found in species that remain within the genus *Turris*. The presence of the venom peptides found in *Purpuraturris* species but not in any *Turris* may suggest that these play a role in aspects of *Purpuraturris* biology divergent from the biology of true *Turris* species. The three divergent branches of the tree found in species of *Turris sensu stricto* in turn, suggests that these may play a functional role exclusive to the biology of members of that genus.

While we cannot definitively conclude that clade II P-like turripeptides are without exception absent from *Turris* species, nor that clade III and IV P-like turripeptides are universally absent from *Purpuraturris* species, by differentiating the bioactivity of a P-like turripeptide representative from each branch of the phylogenetic tree in Figure 5, an understanding of the differences in underlying molecular mechanisms can be achieved more efficiently. A correlation of the bioactivity of the various peptides with the biology of the snails that evolved these venom peptides helps to build a foundation for understanding how a peptide in a particular venom contributes to the biology of that species. Furthermore, details of the effects of the peptide on a target animal (presumably prey, predator or competitor) can connect molecular mechanisms to the systems level and behavior. The distribution of homologous peptides across a particular lineage, in this case, the species in the genus *Turris*, will suggest whether that specific venom peptide is involved in a biological strategy shared widely across the genus *Turris* by many species, or whether a particular type of peptide represents an innovative facet of a subset of species in that lineage. Behavioral observations of the species in a particular lineage may then be correlated with particular venom peptides. For example, phylogenetic analysis of A-superfamily toxins from fish-hunting cone snails revealed that distinct clades of toxins correlate with distinct subtype selectivity profiles at the nicotinic acetylcholine receptor (nAChR) (Puillandre et al., 2010).

### Selection of Venom Peptides for Biological Characterization

The assessment of which peptides in a large venom peptide library should be selected for further analysis can be viewed as a three-step process. The first step is to obtain the library of venom peptide sequences from a lineage of related venomous animals, in this study, the species conventionally assigned to a single genus, *Turris*. The phylogenetic analysis revealed that a group of four species were not congeneric, but in fact, were only distantly related to species correctly assigned to *Turris*. By carrying out the analysis of both groups of species and analyzing the peptide sequences obtained, peptides more closely related to each other could be grouped into discrete clusters. Each cluster likely represents a different underlying mechanism for bioactivity, and analyzing at least one representative of each cluster would be a priority goal.

Some clusters however, are represented by multiple peptides even within the venom of a single species; these may have related molecular targets. If the potential macromolecular targets are of high interest, it may be desirable to investigate the subgroups within each cluster of related peptides. Investigation of dissimilar peptides based on branch lengths in the venom peptide phylogenetic tree may also help prioritize the selection peptides likely to have differences in their bioactivities. This initial phase of analysis is based purely on the peptide sequences initially obtained. Additional input data for developing an experimental strategy are the level of expression of peptides that define a particular cluster and the ability to carry out a thorough proteomic analysis so that the native venom peptide can be compared as comprehensively as possible with a peptide that is either synthesized chemically or produced through recombinant expression. In the case of conoidean venom peptide libraries, this is particularly important because of the frequent presence of posttranslational modifications.

An additional consideration for which peptides to characterize is which venom can be obtained in greater quantity and without negative ecological impact; in addition to the phylogenetic data and the level of expression derived from the transcriptomic data, a proteomic analysis confers significant advantages, and this generally requires a larger venom sample than is required for the other assessments. Thus, in the Thd9.1 cluster, whether it should be the peptide from *T. hidalgoi* or from *T. spectabilis* that is investigated depends on which venom is more easily available. The more abundant venom can be fractionated using the standard HPLC methods to purify individual peptides, and a mass spectrometric analysis with appropriate proteomic bioinformatics should identify peptides in the crude venom related to the Thd9.1 cluster. This analysis is important because it would uncover any posttranslational modifications, which is essential information required before initiating a comprehensive biochemical characterization. Furthermore, if a sufficient amount of venom were available to purify the native venom peptide in large amounts, after the peptide has been chemically synthesized (or produced by recombinant methods), a thorough comparison between the synthetic and the native peptides can be carried out, both with respect to biochemical identity, as well as to demonstrate similar bioactivity. These experiments are greatly facilitated by having larger amounts of native material readily available.

Thus, even before any biochemical work is initiated, the venom peptide library can be better assessed if the following were available: 1. A phylogeny of species in the lineage from which the peptide library was obtained. 2. A tree clustering individual members of the library into groups in order to assess their relatedness, 3. The level of expression of each venom peptide in the library. 4. A proteomic analysis of the venom to identify post-translational modifications and proteolytic cleavage sites, 5. Purified native peptide from venom for subsequent structural and functional comparison to a synthetic analog. Together, these can be used to prioritize which specific peptides will be the leads for chemical synthesis and biological characterization. Assembling these data leads to an informed decision of which peptides in the venom peptide library should be investigated. Since the characterization of each individual peptide is a major commitment of time and resources, this is a key decision point.

Once a significant quantity of the authentic bioactive peptide becomes available, either through chemical synthesis or recombinant expression, assessing potential biomedical applications can then be initiated. Several major factors influence how this assessment should be carried out: which biomedical applications are of the highest priority to the researchers (e.g., cancer applications, pain, neurodegenerative disease), what range of relevant assays are accessible, and the quantity of peptide available for the initial characterization. Since these factors vary considerably, which peptides to focus on for biomedical applications such as drug development will clearly be dictated by the results of the initial characterization. Venom peptides that have a novel activity and that score on an assay that has relevance for specific biomedical applications could then be prioritized for further comprehensive characterization and assessment.

## Methods

### Cloning and sequencing of cDNA Clones Encoding *Turris normandavidsoni* PII and PIII Turripeptide Sequences

RNA isolation and cDNA synthesis were performed as previously described for *Iotyrris olangoensis* (Watkins et al., 2006). First strand cDNA from *Turris normandavidsoni* was used as template to amplify the genes encoding PII and PIII turripeptides. Oligonucleotide primers were designed from untranslated region sequences flanking the open reading frame of PII turripeptide-encoding clones identified from *Iotyrris olangoensis* (Watkins et al., 2006) cDNA to amplify PII turripeptide sequences from *Turris normandavidsoni* (Tnr9.2; Table 1, Supplementary Table S1): 5’ (GAA CCR GCC AGC RAG ATG GG) and 3’ (GCC ATC AGT TGA CAA CAC GTG). Primers to the signal sequence and 3′UTR regions of PIII turripeptide-encoding genes identified from *Iotyrris olangoensis* (Watkins et al., 2006) were used to amplify PIII turritoxin sequences from *Turris normandavidsoni* (Tn9.3 and Tnr9.4; Table 1, Supplementary Table S1): 5’ (GAA CCR GCC AGC RAG) and 3’ (GCC ATC AGT TGA CAA CAC GTG). Polymerase chain reactions were performed with Advantage 2 polymerase Mix as described for mitochondrial gene products. The PCR cycling profiles are as follows: Initial denaturation (95°C, 60s); followed by 40 cycles of denaturation (95°C, 20s); annealing (58°C, 20s) and extension (72°C, 30s).

The resulting PCR products were purified, annealed to pNEB206A vector and transformed into competent cells, as described for mitochondrial gene products.

The nucleic acid sequences of these PII and PIII turritoxin-encoding clones were determined by automated sequencing (Core Sequencing Facility, University of Utah, United States).

### Transcriptome Based Phylogeny

Venom gland transcriptomes were generated as previously described (Li et al., 2018). Briefly, adapter clipping and quality trimming of raw reads were performed using fqtrim software (Version 0.9.4, http://ccb.jhu.edu/software/fqtrim/) and PRINSEQ (Version 0.20.4) (Schmieder and Edwards, 2011). After processing, sequences shorter than 70 bps and those containing more than 5% ambiguous bases (Ns) were discarded. De novo transcriptome assembly was performed using Trinity Version 2.0.5 (Grabherr et al., 2011) with a kmer size for building De Bruijn Graphs of 31, a minimum kmer coverage of 10, and a minimum glue of 10. Assembled transcripts were annotated using Blastx [(NCBI-Blast-2.2.28+, (Altschul et al., 1990)] against conotoxin sequences extracted from the ConoServer (Kaas et al., 2008) and (UniProt Consortium, 2015). The species, number of transcripts, toxins identified, toxin transcript expression values (tpm) and the number of toxin superfamilies identified in each of the 35 transcriptomes used here are listed in Supplementary Table S3. These transcriptomes were assessed using BUSCO analysis (busco:v5.2.2_cv2) (Manni et al., 2021) with the database mollusca_odb10 (5295 entries) and found to be of comparable quality (Supplementary Table S3). Common genes that were shared between all samples were identified using the blast identities of assembled contigs (e < 10^–4^). This set of 66 housekeeping genes (HKG), half of which are ribosomal genes, is listed in Supplementary Table S4, along with Uniprot accession numbers. For each HKG, we aligned the nucleotide sequences from all species using mafft v7.407 (Katoh et al., 2005) (with the “auto” flag). For each alignment file we used the RAxML v8.2.12 (Stamatakis, 2006) program with a GTRGAMMA model (unpartitioned) to determine the “best” tree. Finally, we used Astral (version 5.7.3) (Zhang et al., 2018) to generate a consensus tree from all the individual single-gene trees. The HKG dataset generated for this study is included in the DRYAD repository (DOI): doi:10.5061/dryad.ksn02v751) as fasta files, aligned nt mafft fasta files and newick formatted “best” trees.

The identities of the turrid specimens used for transcriptome-based phylogeny were confirmed using the COI barcode sequence, in the same manner as described in Methods for standard phylogeny. These GenBank accession numbers are listed in Supplementary Table S2 and are consistent with the COI barcode sequences generated for Figure 4 and listed in Supplementary Table S5.

### Phylogenetic Analysis of Turrid Species Using Standard Molecular Markers

Phylogenetic analysis was performed using concatenated 12S and 16S rRNA and the mitochondrial cytochrome oxidase I (COI) barcode sequences for each species as previously described (Nam et al., 2009, Puillandre et al., 2014). GenBank accession numbers for these standard phylogenetic markers, which were used to generate Figure 4, are listed in Supplementary Table S5.

Multiple sequence alignment was performed using MAFFT version 7 (RRID:SCR_011811) (Katoh et al., 2017), maximum likelihood tree reconstruction was performed using IQ tree (RRID:SCR_021163) (Nguyen et al., 2015), consisting of best-fit model TVM+F+I+G4 using ModelFinder (Kalyaanamoorthy et al., 2017) and ultrafast bootstrap approximation (Minh et al., 2013) and the tree was visualized using iTOL version 6.4 (RRID:SCR_018174) (Letunic and Bork, 2021). The alignment file has been included in the supplementary data as Supplementary Data Sheet S1.

### Phylogenetic Analysis of P-like Turritoxins

The sequences of the P-type turripeptide precursors were aligned using the MAFFT multiple sequence alignment program (RRID:SCR_011811) (wasabi.org) (Veidenberg et al., 2015) and refined by eye. Phylogenetic analysis was performed using IQ tree (RRID:SCR_021163) (Nguyen et al., 2015), consisting of best-fit model JTTDCMut+G4 using ModelFinder (Kalyaanamoorthy et al., 2017) and ultrafast bootstrap approximation (Minh et al., 2013) and the tree was visualized using iTOL version 6.4 (RRID:SCR_018174) (Letunic and Bork, 2021). The phylogenetic tree, which is rooted at the midpoint, is shown in Figure 5A. The alignment has been included in Figure 5B. Complete peptide precursors are shown in Supplementary Table S1, predicted mature peptides are shown in Table 1, and relative expression levels, where available, are shown in Figure 5C. The sequences were deposited into the GenBank nucleotide database, accession numbers: OK247621-OK247652.

## Figures and Tables

**Appendix Figure 1:**
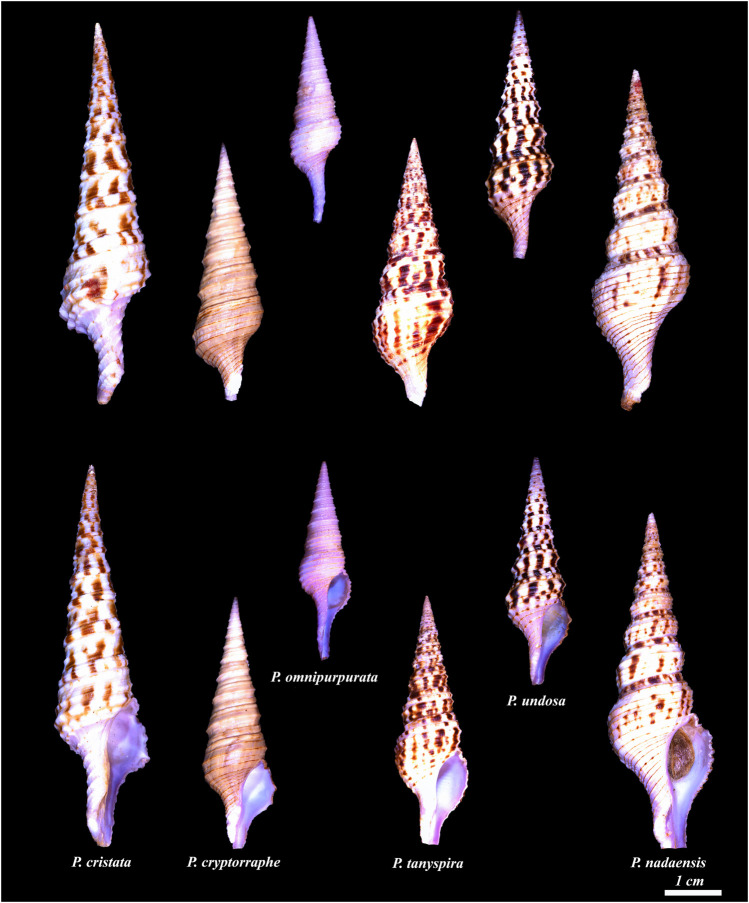
Species in *Purpuraturris gen. nov. *from left to right:* Purpuraturris cristata*;* Purpuraturris cryptorraphe, *type species;* Purpuraturris omnipurpurata*;* Purpuraturris tanyspira*;* Purpuraturris undosa*;* Purpuraturris nadaensis*. Top row shows dorsal view, bottom row shows the apertural view. The shells were photographed individually and the photographs were cropped to maintain the size of the shells relative to each other. The scale bar represents an approximate length of 1 cm. (Photographed by Samuel Espino.)

**Appendix Figure 2:**
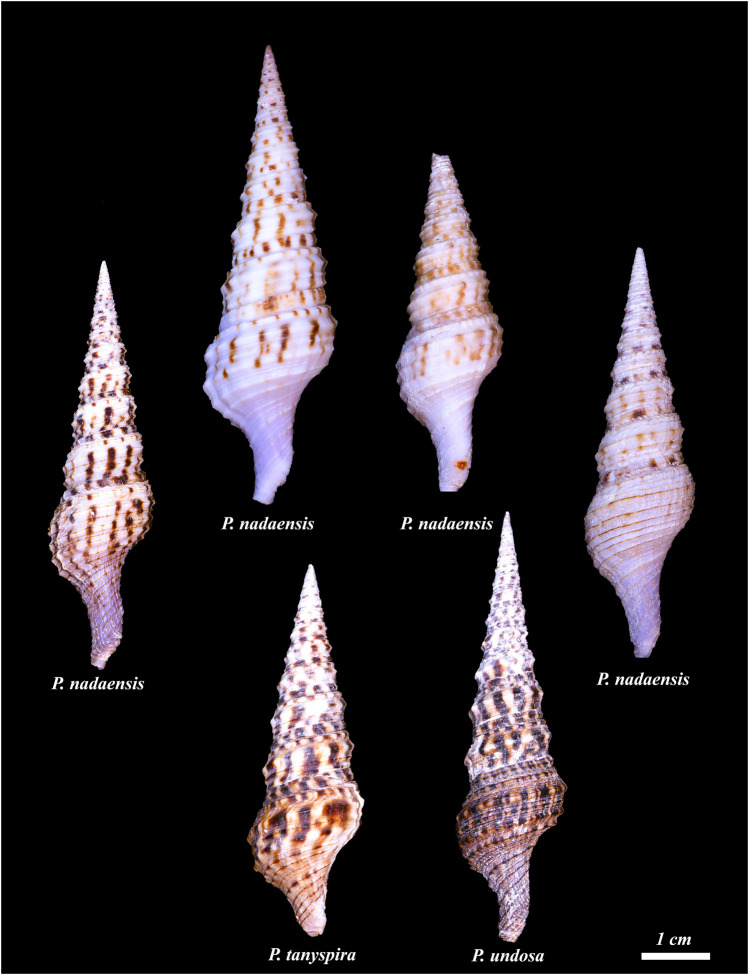
Diverse morphology of specimens assigned to *Purpuraturris nadaensis* and *Purpuraturris undosa*. The figure shows Philippine examples of specimens assigned to either *Purpuraturris nadaensis* or *Purpuraturris undosa*; specimens in the top row were all assigned to *Purpuraturris nadaensis*, and the bottom row to *Purpuraturris undosa*. Note the divergence in morphology, but the overlap between some of the *nadaensis* vs. *undosa* specimens. The figure illustrates the need for a more comprehensive analysis of the morphology of both the shell and other morphological features (e.g., radula), as well as for a molecular analysis of each different morphotype. If specimens from other localities were included, the range of diverse phenotypes would be even greater. The shells were photographed individually and the photographs were cropped to maintain the size of the shells relative to each other. The scale bar represents an approximate length of 1 cm. (Photographed by Samuel Espino.)

## Data Availability

Phylogenetic sequences used in this study have been deposited in GenBank with the indicated accession numbers listed in Supplementary Tables 2,5. Peptide precursor sequences have been deposited into the GenBank nucleotide database, accession numbers OK247621-OK247652. HKG gene sequences obtained from transcriptome data have been included in Dryad: Chase, Kevin; Watkins, Maren (2021), Integrating Venom Peptide Libraries into a Phylogenetic and Broader Biological Framework, Dryad, Dataset, https://doi.org/10.5061/dryad.ksn02v751.

## Appendices

### Appendix 1

**Redefining Generic Assignments of Species formerly in *Turris*. Description of a new genus.** The rationale for proposing a new genus was described in the text; molecular data reveal that a group of species conventionally assigned to the genus *Turris* are only distantly related to true *Turris* (*Turris babylonia*, type species). Since these species are morphologically distinct from all other taxa in Turridae, creation of a new genus is justified. Because of the considerable phylogenetic divergence from *Turris*, creating a subgenus of the *Turris* was not a viable option. We designate *Purpuraturris cryptorraphe* as the type species of the new genus, *Purpuraturris gen. nov*.

**Shell morphology: differences between *Turris* and *Purpuraturris*.** Traditionally, the position of the apertural slit, the thin linear cleft in the margin of the aperture, on the body whorl was used as a definitive character for including species in the genus *Turris*, but the molecular data show that the Turridae with this feature are separable into two groups. A diagnostic difference between the two groups morphologically is the distinctive violet tinge in color of the aperture and spire of *Purpuraturris* spp., which is lacking in all true *Turris* species. A figure of all species formerly assigned to *Turris* that are now reassigned to *Purpuraturris* is shown in Appendix Figure 1.

We repeat the characterization of the genus *Turris* from Kilburn et al., 2012, and add additional descriptive criteria that exclude the species included by Kilburn et al. in *Turris* that are being transferred to the new genus. The defining morphological features of species in the new genus are provided in the formal description.

Genus *Turris*. (Description modified from Kilburn et al., 2012)
Family TURRIDAE H. & A. Adams, 1853
*Turris* Batsch, 1789 — Type species (s.d. Dubois & Bour 2010): *Murex babylonius*, 1758.
Synonym: *Annulaturris* Powell, 1966. Type species (o.d.): *Pleurotoma amicta* E.A. Smith, 1877

DESCRIPTION: Shell medium to large (adult length 40-185 mm), fusiform with high spire and long, unnotched siphonal canal; anal sinus a deep slot situated on a ridge immediately above the peripheral keel; sculpture of spiral cords or ridges, sinus cord often crenulated. Usually with brown spots or stripes. Protoconch usually minute and papilliform, of 2-5 whorls, smooth, later whorls usually axially ribbed; in some species large and bulbous, of 1.5-2.0 smooth whorls. Operculum ungulate, typical of Turrinae.

Radula of duplex marginal teeth, varying considerably in shape, sometimes with a quadrate central tooth bearing a small to large median cusp.

*Turris* is a tropical Indo-West Pacific group. No species of *Turris* occur in the Atlantic Ocean or Mediterranean Sea. The genus *Turris* was distinguished by Powell (1964, 1966) from the very similar genus *Lophiotoma* Casey, 1904, on the basis of the anal sinus being situated on the “special” spiral cord, not on the peripheral cord as in the latter. This convention is followed here, although this difference hinges solely on the relative strength of the two cords, a character not always clearly defined. We follow Li & Li (2007) in leaving the status of *Annulaturris* in abeyance, pending more extensive radula and molecular studies.

All species in *Turris* have a white aperture. A number of species with a purplish aperture or canal, which have been conventionally assigned to this genus do not belong in the redefined genus, but in *Purpuraturris gen. nov*.

*Purpuraturris gen. nov*. — Type species, *Turris cryptorrhaphe*.

DESCRIPTION: Shell medium, fusiform with high spire and medium to short siphonal canal; anal sinus immediately above the peripheral keel. Sculpture consisting of spiral cords or ridges, with the apex of cords often with a dark brown line. Aperture and canal tinted with a purplish hue, but the purplish color fades rapidly. In some specimens, both the siphonal canal and adjacent body whorl have a purple tint. Dark brown stripes that cross the spiral cords are present in some species.

*Purpuraturris* is a tropical group presently known from the Western Pacific to the Indian Ocean.

Speciation in *Purpuraturris* ; geographic distribution. As a group, the species transferred from *Turris* to *Purpuraturris* are deeper and colder water taxa. The best known and most distinctive species is *Purpuraturris cryptorraphe*, which is widely distributed in the Western Pacific. *Purpuraturris cristata* has a massive sutural rib that differentiates it from all other species, a generally higher spire and short siphonal canal. A third distinctive species, which is extremely rare, is *Purpuraturris omnipurpurata*, so far found only in deep water in the Central and Southern Philippines. This species is smaller and more slender with a relatively longer siphonal canal, and spiral ribs usually lined at the apex (similarly to *Purpuraturris cryptorraphe*). A distinctive feature is the absence of markings on any of the spiral ribbons except for the thin brown line at the apex. We believe that specimens assigned to this species that have such markings in their subsutural spiral ribbon are misidentified (in Poppe, Vol. 5, page 582, Plate 1591; specimen #3 from Olango Island is an authentic *Purpuraturris omnipurpurata* specimen, but the specimen illustrated in #4 from Balut Island is, in our opinion, a specimen that belongs to the *Purpuraturris nadaensis* complex). This misidentification is not surprising because of the great variation in the specimens assigned to *Purpuraturris nadaensis*, *Purpuraturris undosa* and overlapping in morphology, *Purpuraturris tanyspira*. The last appears to be separated geographically, being from Southeastern Africa, which suggests that it is probably a different taxon, but all three species are variable, and have overlapping morphological features. A much more comprehensive molecular and morphological characterization is required before definitive assignments can be made at the species level — some of the variations are shown in **Appendix Figure 2**.

Another unsettled question is the proper placement of a number of species that seem intermediate between *Turris* and *Purpuraturris*, morphologically, and of the species that had been assigned to *Annulaturris*. These are all unresolved issues, but the data obtained in this study clearly separate the species that we have reassigned to *Purpuraturris* from those related to *Turris babylonia*, which properly belong to *Turris sensu stricto*.

### Appendix 2

The nomenclature adopted for venom peptides in the family Turridae is based on the convention widely used for disulfide-rich cone snail venoms (Gray et al., 1988; McIntosh et al., 1999), and one that can generally be adapted for use in all conoidean venoms. A parallel and similar nomenclature has been adopted for spider peptide toxins as well (King et al., 2008).

The final name assigned to a given peptide is designed to convey the maximum amount of information.

The toxin name begins with a Greek symbol to identify the molecular target, when known, followed by an uppercase letter to indicate species and a lowercase letter to distinguish between two species beginning with the same letter. Moller et al. have suggested a three-letter code for *Conus* due to a lack of unique combinations using only two letters (Möller et al., 2005). This is followed by a Roman number to indicate the cysteine framework, which is based on the number and arrangement of cysteine residues (Kaas et al., 2012) and finally an uppercase Arabic number to denote the order of peptide discovery. For example, in the cone snail venom peptides ω-Conotoxin GVIA and δ-Conotoxin TxVIA, the Greek letters ω and δ indicate that the first peptide targets voltage-gated calcium channels, while the second peptide blocks the inactivation of voltage-gated sodium channels. The first peptide is from *Conus geographus* (G) and the second is from *Conus textile* (Tx). The Roman numeral VI indicates that they have the same arrangement of cysteines, framework VI (C-C-CC-C-C) and the Arabic numeral A in each indicates that these peptides were the first conotoxins of this framework discovered from each species. Ultimately it is desirable that all peptides be characterized to the point where they can be named to reveal their mechanism of action.

Thus, for the *Purpuraturris* venom peptides, we would adopt a similar nomenclature except that to be able to encompass all conoidean venoms, non-cone snail venom peptides will have an additional letter, but otherwise the rules for nomenclature will be the same. Thus, the first identified Clade II peptide from *Purpuraturris nadaensis* (see manuscript, Figure 5), if targeted to voltage-gated calcium channels would be ultimately designated as ω-Turripeptide PndIXA, while the first peptide from *Purpuraturris cristata* would be ω-Turripeptide PcsIXA. If these were targeted instead to potassium channels, the nomenclature would be κ-Turripeptide TndIXA for a peptide from *Turris normandavidsoni*. The only difference from conopeptide nomenclature is the addition of a preceding letter to denote which genus within the family the peptide is derived from, followed by two lower case letters to denote the species. Thus, the calcium-targeted peptide from *Turris hidalgoi* would be ω-Turripeptide ThdIXA, indicating that this is a peptide from a specific *Turris* species that is targeted to voltage-gated calcium channels and has cysteine framework IX (C-C-C-C-C-C). The advantage of this nomenclature is that it can be readily used for other conoidean families. Thus, the first peptide targeted to calcium channels from the family Drilliidae, for example from the species *Clavus canalicularis*, if it were also were a framework IX peptide, would be called ω-Drillipeptide CcnIXA.

Following the conventional nomenclature established for cone snail venoms (Kaas et al., 2010), we suggest that before the mechanism of the peptide has been elucidated, the three-letter code, followed by an Arabic rather than Roman numeral to indicate the cysteine framework, followed by the clone number be used to identify all peptides with mechanisms that have not been determined (e.g., Tcs9.1, Thd9.8). Lower case roman numerals (ii, iii, iv, etc.) can be added following the clone number to denote minor sequence variation in an otherwise homologous peptide precursor from the same species, such as that arising from allelic variation (e.g., Pcr9.4ii vs Pcr9.4, Tsp9.2ii vs Tsp9.2). The distinction between a clone sequence and an actual biochemically-synthesized peptide could be made by capitalizing the former and using three small letters for the latter. Thus, once the peptide is available biochemically, the clone Thd9.1 is referred to as thd9.1.

## References

[B1] Abdelkrim J. Aznar-Cormano L. Buge B. Fedosov A. Kantor Y. Zaharias P. (2018). Delimiting Species of marine Gastropods (Turridae, Conoidea) Using RAD Sequencing in an Integrative Taxonomy Framework. Mol. Ecol. 27 (22), 4591–4611. 10.1111/mec.14882 30252979

[B2] Altschul S. F. Gish W. Miller W. Myers E. W. Lipman D. J. (1990). Basic Local Alignment Search Tool. J. Mol. Biol. 215 (3), 403–410. 10.1016/s0022-2836(05)80360-2 2231712

[B3] Bouchet P. Kantor Y. I. Sysoev A. Puillandre N. (2011). A New Operational Classification of the Conoidea (Gastropoda). J. Molluscan Stud. 77 (3), 273–308. 10.1093/mollus/eyr017

[B4] Bouchet P. (1990). Turrid Gnera and Mode of Development: the Use and Abuse of Protoconch Morphology. Malacologia 32, 69–77. https://www.biodiversitylibrary.org/page/13146254

[B5] Chen N. Xu S. Zhang Y. Wang F. (2018). Animal Protein Toxins: Origins and Therapeutic Applications. Biophys. Rep. 4 (5), 233–242. 10.1007/s41048-018-0067-x 30533488 PMC6245134

[B6] Fedosov A. Watkins M. Heralde F. M. Corneli P. S. Concepcion G. P. Olivera B. M. (2011). Phylogeny of the Genus Turris: Correlating Molecular Data with Radular Anatomy and Shell Morphology. Mol. Phylogenet. Evol. 59 (2), 263–270. 10.1016/j.ympev.2011.01.019 21352932 PMC4201627

[B7] Grabherr M. G. Haas B. J. Yassour M. Levin J. Z. Thompson D. A. Amit I. (2011). Full-length Transcriptome Assembly from RNA-Seq Data without a Reference Genome. Nat. Biotechnol. 29 (7), 644–652. 10.1038/nbt.1883 21572440 PMC3571712

[B8] Gray W. R. Olivera B. M. Cruz L. J. (1988). PEPTIDE TOXINS FROM VENOMOUS CONUS SNAILS. Annu. Rev. Biochem. 57 (1), 665–700. 10.1146/annurev.bi.57.070188.003313 3052286

[B9] Heralde F. M. Iii Imperial J. Bandyopadhyay P. K. Olivera B. M. Concepcion G. P. Santos A. D. (2008). A Rapidly Diverging Superfamily of Peptide Toxins in Venomous Gemmula Species. Toxicon 51 (5), 890–897. 10.1016/j.toxicon.2007.12.022 18272193 PMC2582027

[B10] Holford M. Puillandre N. Modica M. V. Watkins M. Collin R. Bermingham E. (2009). Correlating Molecular Phylogeny with Venom Apparatus Occurrence in Panamic Auger Snails (Terebridae). PLOS ONE 4 (11), e7667. 10.1371/journal.pone.0007667 19890382 PMC2766622

[B11] Horton T. Kroh A. Ahyong S. Bailly N. Boyko C. B. Brandão S. N. (2021). World Register of Marine Species (WoRMS). WoRMS Editorial Board. Accessed at: https://www.marinespecies.org at VLIZ . (Accessed January 28, 2022). 10.14284/170

[B12] Kaas Q. Westermann J. C. Craik D. J. (2010). Conopeptide Characterization and Classifications: An Analysis Using ConoServer. Toxicon 55 (8), 1491–1509. 10.1016/j.toxicon.2010.03.002 20211197

[B13] Kaas Q. Westermann J.-C. Halai R. Wang C. K. L. Craik D. J. (2008). ConoServer, a Database for Conopeptide Sequences and Structures. Bioinformatics 24 (3), 445–446. 10.1093/bioinformatics/btm596 18065428

[B14] Kaas Q. Yu R. Jin A.-H. Dutertre S. Craik D. J. (2012). ConoServer: Updated Content, Knowledge, and Discovery Tools in the Conopeptide Database. Nucleic Acids Res. 40, D325–D330. 10.1093/nar/gkr886 22058133 PMC3245185

[B15] Kalyaanamoorthy S. Minh B. Q. Wong T. K. F. von Haeseler A. Jermiin L. S. (2017). ModelFinder: Fast Model Selection for Accurate Phylogenetic Estimates. Nat. Methods 14 (6), 587–589. 10.1038/nmeth.4285 28481363 PMC5453245

[B16] Katoh K. Kuma K. Toh H. Miyata T. (2005). MAFFT Version 5: Improvement in Accuracy of Multiple Sequence Alignment. Nucleic Acids Res. 33 (2), 511–518. 10.1093/nar/gki198 15661851 PMC548345

[B17] Katoh K. Rozewicki J. Yamada K. D. (2017). MAFFT Online Service: Multiple Sequence Alignment, Interactive Sequence Choice and Visualization. Brief. Bioinform. 20 (4), 1160–1166. 10.1093/bib/bbx108 PMC6781576 28968734

[B44] Kilburn R. N. Fedosov A. E. Olivera B. M. (2012). Revision of the Genus *Turris* (Gastropoda: Conoidea: Turridae) with the Description of Six New Species. Zootaxa 3244 (1), 1–58. 23847408 PMC3705779

[B18] King G. F. Gentz M. C. Escoubas P. Nicholson G. M. (2008). A Rational Nomenclature for Naming Peptide Toxins from Spiders and Other Venomous Animals. Toxicon 52 (2), 264–276. 10.1016/j.toxicon.2008.05.020 18619481

[B19] Letunic I. Bork P. (2021). Interactive Tree of Life (iTOL) V5: an Online Tool for Phylogenetic Tree Display and Annotation. Nucleic Acids Res. 49 (W1), W293–W296. 10.1093/nar/gkab301 33885785 PMC8265157

[B20] Lewis R. J. Garcia M. L. (2003). Therapeutic Potential of Venom Peptides. Nat. Rev. Drug Discov. 2 (10), 790–802. 10.1038/nrd1197 14526382

[B45] Li B. Li X. (2007). An Account of the Genus Turris Species (Mollusca: Gastropoda: Turridae) From the East and South China Seas. Zootaxa 1397, 63–68.

[B21] Li Q. Barghi N. Lu A. Fedosov A. E. Bandyopadhyay P. K. Lluisma A. O. (2017). Divergence of the Venom Exogene Repertoire in Two Sister Species of Turriconus. Genome Biol. Evol. 9 (9), 2211–2225. 10.1093/gbe/evx157 28922871 PMC5604253

[B22] Li Q. Watkins M. Robinson S. Safavi-Hemami H. Yandell M. (2018). Discovery of Novel Conotoxin Candidates Using Machine Learning. Toxins 10 (12), 503. 10.3390/toxins10120503 PMC6315676 30513724

[B23] Lu A. Watkins M. Li Q. Robinson S. D. Concepcion G. P. Yandell M. (2020). Transcriptomic Profiling Reveals Extraordinary Diversity of Venom Peptides in Unexplored Predatory Gastropods of the Genus Clavus. Genome Biol. Evol. 12 (5), 684–700. 10.1093/gbe/evaa083 32333764 PMC7259678

[B24] Manni M. Berkeley M. R. Seppey M. Simão F. A. Zdobnov E. M. (2021). BUSCO Update: Novel and Streamlined Workflows along with Broader and Deeper Phylogenetic Coverage for Scoring of Eukaryotic, Prokaryotic, and Viral Genomes. Mol. Biol. Evol. 38 (10), 4647–4654. 10.1093/molbev/msab199 34320186 PMC8476166

[B25] McIntosh J. M. Santos A. D. Olivera B. M. (1999). Conus Peptides Targeted to Specific Nicotinic Acetylcholine Receptor Subtypes. Annu. Rev. Biochem. 68 (1), 59–88. 10.1146/annurev.biochem.68.1.59 10872444

[B26] Minh B. Q. Nguyen M. A. T. von Haeseler A. (2013). Ultrafast Approximation for Phylogenetic Bootstrap. Mol. Biol. Evol. 30 (5), 1188–1195. 10.1093/molbev/mst024 23418397 PMC3670741

[B27] Möller C. Rahmankhah S. Lauer-Fields J. Bubis J. Fields G. B. Marí F. (2005). A Novel Conotoxin Framework with a Helix−Loop−Helix (Cs α/α) Fold. Biochemistry 44 (49), 15986–15996. 10.1021/bi0511181 16331958

[B46] Nam H. H. Corneli P. S. Watkins M. Olivera B. Bandyopadhyay P. (2009). Multiple Genes Elucidate the Evolution of Venomous Snail-Hunting Conus Species. Mol. Phylogenet. Evol. 53 (3), 645–652. 10.1016/j.ympev.2009.07.013 19616106

[B28] Nguyen L.-T. Schmidt H. A. von Haeseler A. Minh B. Q. (2015). IQ-TREE: a Fast and Effective Stochastic Algorithm for Estimating Maximum-Likelihood Phylogenies. Mol. Biol. Evol. 32 (1), 268–274. 10.1093/molbev/msu300 25371430 PMC4271533

[B29] Olivera B. M. Seronay R. A. Fedosov A. E. (2010). Turris Babylonia; Re-evaluation of a Species Complex and Description of Turris Assyria, New Species. Philipp Sci. Lett. 3 (1), 20107. 23133790 PMC3488452

[B30] Olivera B. M. Showers Corneli P. Watkins M. Fedosov A. (2014). Biodiversity of Cone Snails and Other Venomous Marine Gastropods: Evolutionary Success through Neuropharmacology. Annu. Rev. Anim. Biosci. 2 (1), 487–513. 10.1146/annurev-animal-022513-114124 25384153

[B31] Pennington M. W. Czerwinski A. Norton R. S. (2018). Peptide Therapeutics from Venom: Current Status and Potential. Bioorg. Med. Chem. 26 (10), 2738–2758. 10.1016/j.bmc.2017.09.029 28988749

[B47] Powell A. W. B. (1964). The Family Turridae in the Indo-Pacific. Part 1. The Subfamily Turrinae. Indo-Pacific Mollusca. 1, 227–346.

[B48] Powell A. W. B. (1966). The Molluscan Families Speightiidae And Turridae. An Evaluation of the Valid Taxa Both Recent and Fossil, With Lists of Characteristic Species. Bulletin of the Auckland Institute and Museum 5, 1–184.

[B32] Puillandre N. Bouchet P. Duda T. F. Jr. Kauferstein S. Kohn A. J. Olivera B. M. (2014). Molecular Phylogeny and Evolution of the Cone Snails (Gastropoda, Conoidea). Mol. Phylogenet. Evol. 78, 290–303. 10.1016/j.ympev.2014.05.023 24878223 PMC5556946

[B33] Puillandre N. Samadi S. Boisselier M.-C. Sysoev A. V. Kantor Y. I. Cruaud C. (2008). Starting to Unravel the Toxoglossan Knot: Molecular Phylogeny of the “Turrids” (Neogastropoda: Conoidea). Mol. Phylogenet. Evol. 47 (3), 1122–1134. 10.1016/j.ympev.2007.11.007 18180170

[B34] Puillandre N. Watkins M. Olivera B. M. (2010). Evolution of Conus Peptide Genes: Duplication and Positive Selection in the A-Superfamily. J. Mol. Evol. 70 (2), 190–202. 10.1007/s00239-010-9321-7 20143226 PMC2943986

[B35] Robinson S. D. Li Q. Lu A. Bandyopadhyay P. K. Yandell M. Olivera B. M. (2017). The Venom Repertoire of Conus Gloriamaris (Chemnitz, 1777), the Glory of the Sea. Mar. Drugs 15 (5), 145. 10.3390/md15050145 PMC5450551 28531118

[B36] Robinson S. Norton R. (2014). Conotoxin Gene Superfamilies. Mar. Drugs 12 (12), 6058–6101. 10.3390/md12126058 25522317 PMC4278219

[B37] Safavi-Hemami H. Lu A. Li Q. Fedosov A. E. Biggs J. Showers Corneli P. (2016). Venom Insulins of Cone Snails Diversify Rapidly and Track Prey Taxa. Mol. Biol. Evol. 33 (11), 2924–2934. 10.1093/molbev/msw174 27524826 PMC5062327

[B38] Schmieder R. Edwards R. (2011). Quality Control and Preprocessing of Metagenomic Datasets. Bioinformatics 27 (6), 863–864. 10.1093/bioinformatics/btr026 21278185 PMC3051327

[B39] Stamatakis A. (2006). RAxML-VI-HPC: Maximum Likelihood-Based Phylogenetic Analyses with Thousands of Taxa and Mixed Models. Bioinformatics 22 (21), 2688–2690. 10.1093/bioinformatics/btl446 16928733

[B40] UniProt Consortium

[B41] Veidenberg A. Medlar A. Löytynoja A. (2015). Wasabi: An Integrated Platform for Evolutionary Sequence Analysis and Data Visualization. Mol. Biol. Evol. 33 (4), 1126–1130. 10.1093/molbev/msv333 26635364

[B42] Watkins M. Hillyard D. R. Olivera B. M. (2006). Genes Expressed in a Turrid Venom Duct: Divergence and Similarity to Conotoxins. J. Mol. Evol. 62 (3), 247–256. 10.1007/s00239-005-0010-x 16477526

[B43] Zhang C. Rabiee M. Sayyari E. Mirarab S. (2018). ASTRAL-III: Polynomial Time Species Tree Reconstruction from Partially Resolved Gene Trees. BMC Bioinformatics 19 (6), 153. 10.1186/s12859-018-2129-y 29745866 PMC5998893

