# Supplementary material for: Integrating Venom Peptide Libraries Into a Phylogenetic and Broader Biological Framework
Source: Front Mol Biosci. 2022 Feb 21;9:784419. doi: 10.3389/fmolb.2022.784419 (PMC8899473; doi:10.3389/fmolb.2022.784419)
Supplement: Supplementary file 1 [file Table1.DOCX]

**Supplementary Table 1.** P-like turripeptide precursors. Turripeptides indicated by an asterisk were identified from pcr or sequencing of cDNA libraries. Turripeptides indicated by a double asterisk were identifed from a transcriptome other than that used for the neutral marker phylogeny. The sequences are separated into two groups, P-like turripeptide superfamily PII (Clades I and II, Figure 5) and P-like turripeptide superfamily PIII (Clades III and IV, Figure 5) and further described in Methods.

**P-like turripeptide superfamily PII**

| **Species** | **Toxin name** | **Complete prepropeptide sequence** |
| --- | --- | --- |
| *Turris bablyonia* | Tba9.3* | ﻿MGFSILLTVVALLLTSFMSTDATPVDQAKRRNGPGTRIWSR  DACPGNEAKCFSTECTNPSSHGYDSQECQDACQYVWDYCSEE* |
| *Turris guidopoppei* | Tgd9.1 | ﻿MGFYILLTVVALLLTSFMSTDATPVDQAKRSTIRKNGPGTRIWSR  DACPEYEAKCFSTECTDEDSDGYDSPECQAACQYVWDHCSED* |
| *Turris hidalgoi* | Thd9.5** | MGFYILLTVVALLLTSFLSTDATSVDQAKRKNGPGTRIWSR  DACPENKVKCFSTECMNLESDGYDSAECQAACQYVYDQCPEE* |
| *Turris normandavidsoni* | Tnr9.2* | ﻿MGFYILLTVVVLLLTSFKSTDATPVDQAKRKNGPGTRIWSR  DACPENEAKCYSTECTNQQADGYDSSECQAACQYVWNHCSYE* |
| *Purpuraturris nadaensis* | Pna9.13** | ﻿MRFSVLLIVALLLAYLMSINARDQAEEKRSTMKKGGHAIMIMPR  DLCDESLANCTSSSCQAELENENGSSACTEACDYWVANCQEK* |
| *Purpuraturris cryptorrhaphe* | Pcr9.4ii | ﻿MRFGVLLLIVALLLAYLMSINARDQAEEKRSTMKKGGHAIMIMPR  DLCDEYLENCTSPYCQEQSNIQNGSSACNEACNYWDKNCRTPDEEQ* |
| *Purpuraturris cryptorrhaphe* | Pcr9.4 | ﻿MRFGVLLLIVALLLAYLMSINARDQAEEKRSTMKKGGHAIMIMPR  DLCDEYLENCTSPYCQEQSNIQNGDGACNEACNYWDKNCRTPDEEQ* |
| *Purpuraturris nadaensis* | Pna9.11 | ﻿MRFSVLLIVALLLAYLMSIDARGQAKRKRSTMKKGTHGIMIMPRD  ACEDSLEECTSEFCIEQSATQNGNAACNSACNYWYHNCQEK* |
| *Purpuraturris nadaensis* | Pna9.12* | ﻿MRFSVLLIVALLLAYLMSIDTRGQAKRKRSTMKKGRHAIMIMPR  DACEDHLEYCTSEFCIEQSYIQNGNATCQNACYDWYQNCQ* |
| *Purpuraturris nadaensis* | Pna9.31** | ﻿MRFSVLLIVALLLAYLMSINARGQAKRKRSTMKKGTHGIMIMPR  DACEDNLEDCTSEFCIEQSATQNGNAACNSACSDWYHNCQ* |
| *Purpuraturris cristata* | Pcs9.1 | ﻿MRFNILLIVALLLILHMSLNATYGGQAPWKRSAMRKARRHGIIMPR  DACESNLETCTSLECMTELQTQTASPACNNACSNYTSNC* |
| *Purpuraturris nadaensis* | Pna9.29 | ﻿MRFSVLLIVALLLAYLMSIDARDQAEEKRSTMKKDRHGILIMPR  DACQETFEYCTSDFCMEELEYEDANVTCVDACNIWLANCQ* |
| *Purpuraturris nadaensis* | Pna9.10* | ﻿MRFSVLLIVALLLAYLMSIVARGQAKRKRSTMKKGGHGIMIMPR  DVCEENRVHCTSPFCQEELEYEDANVTCVDACNIWLANCQ* |
| *Purpuraturris nadaensis* | Pna9.10ii | ﻿MRFSVLLIVALLLAYLMSIDARGQAKRKRSTMKKGGHGIMIMPR  DVCEENRVHCTSPFCQEELEYEDANVTCVDACNIWLANCQ* |
| *Purpuraturris undosa* | Pun9.4 | ﻿MRFSVLLIVALLLAYLMSIDARGQAKRKRSTMKKGGHGIMIMPR  DVCEENRVHCTSPFCQEELEYEDANVTCVDACNIWLANCQ* |
| *Purpuraturris nadaensis* | Pna9.2ii** | ﻿MRFSVLLIVALLLAYLMSIDARDQAEEKRSTMKKGGHGIMIMPR  DVCEDNRVYCTSPFCQEELEYEDANVTCVDACNIWLANCQEK* |
| *Purpuraturris nadaensis* | Pna9.2* | ﻿MRFSVLLIVALLLAYLMSIDARDQAEEKRSTMKKGGHGIMIMPR  DVCEDNRVYCTSPFCQEELEYEDANVTCVDACNIWFANCQEK* |

**P-like turripeptide superfamily PIII**

| **Species** | **Toxin name** | **Complete prepropeptide sequence** |
| --- | --- | --- |
| *Turris hidalgoi* | Thd9.1 | ﻿MKFHLLTLALFLTAVMSIGATPINLVKNERSAMKPLMKMIR  QNNNCGCGSADVGRNCPGFGFCSDGTCSVSNTCEF* |
| *Turris spectabilis* | Tsp9.1 | ﻿MKCHFLTLALFLTAVMSIGATPISLVKNERSAMKPLMKMIR  QNNNCGCASTDVGKPCPGSGLCGSGTCSVLNTCDFE* |
| *Turris spectabilis* | Tsp9.2ii | ﻿MKCHFLTLALFLTAVMSIGATPISLVKNERSAMKPFMKMIR  NNNNCGCGSTDVGQPCPGYGLCNDGICSALNTCDFSVN* |
| *Turris spectabilis* | Tsp9.2 | ﻿MKCHFLTLALFLTAVMSIGATPISLVKNERSAMKPFMKMIR  NNNNCGCGSTDVGQPCPGYGLCNDGICSALNTCDFEIKR* |
| *Turris guidopoppei* | Tgd9.5 | ﻿MKFHLLTLALFLTAVMSIGATPINLMKNERSAVKPRMKMIR  QSNCGCGNTNVGLPCPGTGLCSGICSIAHTCESVDLKR* |
| *Turris hidalgoi* | Thd9.2 | ﻿MKFQLLTLALFLTVVMSIGATPINLVKNERSAMNPLTKMIR  QNCGCGNTGVDQPCPGSGMCINGICTVAYTCKTKR* |
| *Turris babylonia* | Tba9.4* | ﻿﻿MKFHLLTLALFLTAVMSIGATPISLVKNERSAMNPLMKMIR  QNNCGCGHINVNQPCPESGSGCSGGYYSSAHTCEY* |
| *Turris babylonia* | Tba9.5* | ﻿MKFHLLTLTLFLTAVMSIGATPIILVKNERSAMNPLMKMIR  QNNCGCSNRNAGYPCPESSNECSGGVCSLAHTCEL* |
| *Turris hidalgoi* | Thd9.10 | ﻿MRFLALPLLLTAVSSIDTTPINPMEQERSAMPSFLKTLLLKR  WYDCTCEGVEVGSTCSGNNCAAVCRSDGGCWF* |
| *Turris normandavidsoni* | Tnr9.4* | ﻿MGFLTLVLLLTAVMSTDTTPINPVEQERSAMPSFLKILLLQRR  DDCSCEGVEVDSTCSGNSCAAICRSDGRCWI* |
| *Turris guidopoppei* | Tgd9.6 | ﻿MRFLTLALLLTAVMSTDTTPINPVEQKRSAMPSFMKTLLLSR  WYDCTCVEVGSTCSGNSCAAVCRSDVGCWI* |
| *Turris guidopoppei* | Tgd9.15 | ﻿MRFLTLALLLTAVMSTDTTPVNPVEQKRSAMPSFLKTLLLQR  LYDCTCEGVEVGSTCSGNSCAAVCRSDGGCWI* |
| *Turris normandavidsoni* | Tnr9.3ii | ﻿MRFLTLTLLLTAVMSIATTPINPVEQERSAMSSFLKTFLLLQRR  HGCSCEGVEVGSTCSGNDCAAVCRSDGGCWIST* |
| *Turris normandavidsoni* | Tnr9.3* | ﻿MGFLTLTLLLTAVMSIDTTPINPVEQERSAMSSFLKTFLLLQRR  HGCSCEGVEVGSTCSGNDCAAVCRSDGGCWIST* |
| *Turris dollyae* | Tdo9.4 | ﻿﻿MRFLTLTLLLTAVMSIDTTPINPVEQERSAMSSFLKTFLLLQRR  HGCSCEGVEVGSTCAGNDCAAVCRSDGGCWIST* |
