# Supplementary material for: Integrating Venom Peptide Libraries Into a Phylogenetic and Broader Biological Framework
Source: Front Mol Biosci. 2022 Feb 21;9:784419. doi: 10.3389/fmolb.2022.784419 (PMC8899473; doi:10.3389/fmolb.2022.784419)
Supplement: Supplementary file 3 [file Table2.DOCX]

**Supplementary Table 2.** List of turrid species analyzed in transcriptomes. Specimen identity confirmed with COI barcode sequences.

| **Genus** | **Species** | **Location** | **Collection date** | **Inventory #** | **COI confirmation** |
| --- | --- | --- | --- | --- | --- |
| *Gemmula* | *lisajoni* | Sogod | Dec-2018 | 1856 | not sampled |
| *Gemmula* | *sogodensis* | Sogod | Dec-2015 | 1857 | MZ927115 |
| *Iotyrris* | *cinguligfera* | Sogod | Jun-2018 | 1801 | MZ927116 |
| *Iotyrris* | *cingulifera*2 | Cebu | Dec-2018 | - | not sampled |
| *Iotyrris* | *musivum* | Cebu | Jan-2019 | 1811 | MZ927117 |
| *Lophiotoma* | *abbreviata* | Philippines | Sep-2011 | 1448 | not sampled |
| *Lophiotoma* | *picturata* | Cebu | Jan-2019 | 1813 | MZ927118 |
| *Purpuraturris* | *cristata*1 | Caw-oy | Sep-2009 | 1014 | GU299972 |
| *Purpuraturris* | *cristata*2 | Sogod | Jun-2018 | 1803 | MZ927120 |
| *Purpuraturris* | *cryptorrhaphe* | Philippines | Aug-2010 | 1224 | MZ927121 |
| *Purpuraturris* | *nadaensis*1 | Sogod | Nov-2019 | 1826 | MZ927122 |
| *Purpuraturris* | *nadaensis*2 | Sogod | Nov-2019 | 1827 | MZ927123 |
| *Purpuraturris* | *nadaensis*3 | Sogod | May-2006 | 590 | GU299981 |
| *Purpuraturris* | *undosa* | Caw-oy | Jan-2013 | 1641 | MZ927119 |
| *Turris* | *dollyae* | Sogod | Dec-2015 | 1854 | MZ927125 |
| *Turris* | *guidopoppei* | Sogod | Jun-2018 | 1804 | MZ927126 |
| *Turris* | *hidalgoi*1 | Cebu | Jan-2019 | - | not sampled |
| *Turris* | *hidalgoi*2 | Caw-oy | Jan-2013 | 1638 | MZ927127 |
| *Turris* | *normandavidsoni* | Sogod | Dec-2015 | 1858 | MZ927128 |
| *Turris* | *spectabilis* | Sogod | Jun-2018 | 1805 | MZ927129 |
| *Unedogemmula* | *tayabasensis* | Sogod | Jun-2018 | 1806 | MZ927130 |
| *Unedogemmula* | *unedo* | Sogod | Jan-2019 | 1819 | not sampled |
