# Supplementary material for: Integrating Venom Peptide Libraries Into a Phylogenetic and Broader Biological Framework
Source: Front Mol Biosci. 2022 Feb 21;9:784419. doi: 10.3389/fmolb.2022.784419 (PMC8899473; doi:10.3389/fmolb.2022.784419)
Supplement: Supplementary file 4 [file Table3.docx]

**Supplementary Table 3.** Genbank accession numbers and publication references of turrid species used for standard phylogeny in this study.

| **Genus/species** | **Isolate** | **12S** | **16S** | **COI** | **References** |
| --- | --- | --- | --- | --- | --- |
| *Gemmula albina* | MW287 | GU300019 | GU345769 | GU299986 | Fedosov et al., 2011(1) |
| *Gemmula ambara* | MW442 | KF915078 | KF915092 | KF915103 | Watkins, M. (unpublished) |
| *Gemmula diomedea* | MW515 | GU300027 | GU345775 | GU299994 | Fedosov et al., 2011(1) |
| *Gemmula hindsiana* | Ghinds07030117 | KM218646 | not sampled | KM218743 | Todd, JA. & Rawlings, TA, 2014(2) |
| *Gemmula kieneri* | MW261 | KF915082 | KF915093 | KF915105 | Watkins, M. (unpublished) |
| *Gemmula lisajoni* | MW757 | GU300028 | GU345776 | GU299995 | Fedosov et al., 2011(1) |
| *Gemmula monilifera* | MW432 | GU300029 | GU345777 | GU299996 | Fedosov et al., 2011(1) |
| *Gemmula rosario* | FHeralde | EF467336 | GU434135 | not sampled | Heralde et al., 2007(3), Heralde et al., 2010(4) |
| *Gemmula sogodensis* | MW903 | EF467337 | GU434132 | KF915107 | Heralde et al., 2007(3), Heralde et al., 2010(4), Watkins, M.(unpublished) |
| *Gemmula speciosa* | MW434 | GU300030 | GU345778 | GU299997 | Fedosov et al., 2011(1) |
| *Iotyrris cerithiformis* | MW351 | EU682298 | EU682307 | GU299987 | Nam et al., 2009(5), Fedosov et al., 2011(1) |
| *Iotyrris cingulifera* | MW1355 | MZ667645 | MZ667640 | MZ663831 | This publication |
| *Iotyrris olangoensis* | MW315 | FJ868123 | FJ868137 | GU299990 | Biggs, et al. 2010(6), Fedosov et al., 2011(1) |
| *Lophiotoma abbreviata* | MW924 | MZ667646 | MZ667641 | MZ663832 | This publication |
| *Lophiotoma acuta* | MW513 | GU300018 | GU345768 | GU299985 | Fedosov et al., 2011(1) |
| *Lophiotoma brevicaudata* | MW545 | MZ667647 | MZ667642 | MZ663833 | This publication |
| *Lophiotoma jickelli* | MW230 | GU300021 | GU345770 | GU299988 | Fedosov et al., 2011(1) |
| *Lophiotoma picturata* | MW587 | KF915085 | KF915096 | KF915109 | Watkins, M. (unpublished) |
| *Lophiotoma polytropa* | MW447 | EF467347 | GU471190 | EU820480 | Heralde et al., 2007(3), Heralde et al., 2010(4), Puillandre et al., 2012(7){Puillandre, 2012 #457} |
| *Polystira albida* | MW1575 | EF467350 | JF276963 | JF276942 | Heralde et al., 2007(3), Puillandre et al., 2011(8) |
| *Polystira picta* | MW695 | GU827608 | GU827609 | KF915100 | Heralde et al., 2010(4), Watkins, M. (unpublished) |
| *(Purpura)turris cristata* | MW1014 | GU300005 | GU345755 | GU299972 | Fedosov et al., 2011(1) |
| *(Purpura)turris cryptorrhaphe* | MW1016 | GU300008 | GU345758 | GU299975 | Fedosov et al., 2011(1) |
| *(Purpura)turris nadaensis* | MW1022 | GU300010 | GU345760 | GU299977 | Fedosov et al., 2011(1) |
| *(Purpura)turris undosa* | MW1020 | GU300013 | GU345763 | GU299980 | Fedosov et al., 2011(1) |
| *Turridrupa bijubata* | MW1723 | MZ667648 | MZ667643 | MZ663834 | This publication |
| *Turridrupa elongata* | MW700 | GU300032 | GU345780 | GU299999 | Heralde et al., 2010(4), Fedosov et al., 2011(1) |
| *Turridrupa jubata* | MW3159 | KF915077 | KF915091 | KF915102 | Watkins, M. (unpublished) |
| *Turridrupa neojubata* | MW831 | JF823582 | JF823604 | JF823622 | Cabang et al., 2011(9) |
| *Turris babylonia* | MW1010 | GU300002 | GU345752 | GU299969 | Fedosov et al., 2011(1) |
| *Turris dollyae* | MW845 | GU300009 | GU345759 | GU299976 | Fedosov et al., 2011(1) |
| *Turris guidopoppei* | MW1018 | KF915073 | KF915086 | KF915097 | Watkins, M. (unpublished) |
| *Turris grandis* | MW761 | GU300004 | GU345754 | GU299971 | Fedosov et al., 2011(1) |
| *Turris hidalgoi* | MW1029 | KF915080 | KF915087 | KF915098 | Watkins, M. (unpublished) |
| *Turris normandavidsoni* | MW1025 | GU300016 | GU345766 | GU299983 | Fedosov et al., 2011(1) |
| *Turris spectabilis* | MW600 | GU300017 | GU345767 | GU299984 | Fedosov et al., 2011(1) |
| *Unedogemmula bisaya* | MW826 | KF915084 | KF915095 | EU820664 | Watkins, M. (unpublished), Puillandre et al., 2012(7) |
| *Unedogemmula indica* | MW263 | EF467343 | GU827612 | MZ663835 | Heralde et al., 2007(3), Heralde et al., 2010(4), this publication |
| *Unedogemmula panglaoensis* | MW409 | EF467346 | GU827613 | EU820642 | Heralde et al., 2007(3), Heralde et al., 2010(4), Puillandre et al., 2012(7) |
| *Unedogemmula tayabasensis* | FHeralde | EF467348 | GU345772 | GU299991 | Heralde et al., 2007(3), Fedosov et al., 2011(1) |
| *Unedogemmula unedo* | MW228 | EF467349 | MZ667644 | not sampled | Heralde et al., 2007(3), this publication |
| *Xenuroturris legitima* | MW240 | EF467342 | GU585765 | KF915108 | Heralde et al., 2007(3), Heralde et al., 2010(4), Watkins, M. (unpublished) |
| *Xenuroturris millipunctata* | MW530 | GU300022 | GU345771 | GU299989 | Fedosov et al., 2011(1) |

**References for Supplementary Table 3**

Biggs, J.S., Watkins, M., Puillandre, N., Ownby, J.P., Lopez-Vera, E., Christensen, S., et al. (2010). Evolution of Conus peptide toxins: analysis of Conus californicus Reeve, 1844. *Mol Phylogenet Evol* 56(1)**,** 1-12. doi: 10.1016/j.ympev.2010.03.029.

Cabang, A.B., Imperial, J.S., Gajewiak, J., Watkins, M., Corneli, P.S., Olivera, B.M., et al. (2011). Characterization of a venom peptide from a crassispirid gastropod. *Toxicon* 58(8)**,** 672-680. doi: 10.1016/j.toxicon.2011.09.001.

Fedosov, A., Watkins, M., Heralde, F.M., Corneli, P.S., Concepcion, G.P., and Olivera, B.M. (2011). Phylogeny of the genus Turris: Correlating molecular data with radular anatomy and shell morphology. *Molecular Phylogenetics and Evolution* 59(2)**,** 263-270. doi: <https://doi.org/10.1016/j.ympev.2011.01.019>.

Heralde, F.M., 3rd, Kantor, Y.I., Astilla, M.A.Q., Lluisma, A.O., Geronimo, R., Aliño, P.M., et al. (2010). THE INDO-PACIFIC GEMMULA SPECIES IN THE SUBFAMILY TURRINAE: ASPECTS OF FIELD DISTRIBUTION, MOLECULAR PHYLOGENY, RADULAR ANATOMY AND FEEDING ECOLOGY. *Philippine science letters* 3(1)**,** 20105.

Heralde III, F.M., Watkins, M., Ownby, J.-P., Bandyopadhyay, P. K., Santos, A. D., Concepcion, G. P. , & Olivera, B. M. (2007). Moluecular phylogeny of some Indo-Pacific genera in the subfmily Turrinae H. Adams and A. Adams, 1853 (1838) (Gastropoda: Neogastropoda). *Nautilus (Philadelphia)* 121**,** 131-138.

Nam, H.H., Corneli, P.S., Watkins, M., Olivera, B., and Bandyopadhyay, P. (2009). Multiple genes elucidate the evolution of venomous snail-hunting Conus species. *Mol Phylogenet Evol* 53(3)**,** 645-652. doi: 10.1016/j.ympev.2009.07.013.

Puillandre, N., Kantor, Y., Sysoev, A., Couloux, A., Meyer, C., Rawlings, T., et al. (2011). The dragon tamed? A molecular phylogeny of the Conoidea (Gastropoda). *Journal of Molluscan Studies* 77**,** 259-272. doi: 10.1093/mollus/eyr015.

Puillandre, N., Modica, M.V., Zhang, Y., Sirovich, L., Boisselier, M.C., Cruaud, C., et al. (2012). Large-scale species delimitation method for hyperdiverse groups. *Mol Ecol* 21(11)**,** 2671-2691. doi: 10.1111/j.1365-294X.2012.05559.x.

Todd, J.A., and Rawlings, T.A. (2014). A review of the Polystira clade--the Neotropic's largest marine gastropod radiation (Neogastropoda: Conoidea: Turridae sensu stricto). *Zootaxa* 3884(5)**,** 445-491. doi: 10.11646/zootaxa.3884.5.5.
